# The Dmipy Toolbox: Diffusion MRI Multi-Compartment Modeling and Microstructure Recovery Made Easy

**DOI:** 10.3389/fninf.2019.00064

**Published:** 2019-10-15

**Authors:** Rutger H. J. Fick, Demian Wassermann, Rachid Deriche

**Affiliations:** ^1^TheraPanacea, Paris, France; ^2^Inria Sophia Antipolis-Méditerranée, Université Côte d'Azur, Sophia Antipolis, France; ^3^INRIA, CEA, Université Paris-Saclay, Paris, France

**Keywords:** diffusion MRI, multi-compartment modeling, microstructure estimation, reproducible research, neuroimaging, optimization, open-source, PGSE

## Abstract

Non-invasive estimation of brain microstructure features using diffusion MRI (dMRI)—known as Microstructure Imaging—has become an increasingly diverse and complicated field over the last decades. Multi-compartment (MC)-models, representing the measured diffusion signal as a linear combination of signal models of distinct tissue types, have been developed in many forms to estimate these features. However, a generalized implementation of MC-modeling as a whole, providing deeper insights in its capabilities, remains missing. To address this fact, we present Diffusion Microstructure Imaging in Python (Dmipy), an open-source toolbox implementing PGSE-based MC-modeling in its most general form. Dmipy allows on-the-fly implementation, signal modeling, and optimization of any user-defined MC-model, for any PGSE acquisition scheme. Dmipy follows a “building block”-based philosophy to Microstructure Imaging, meaning MC-models are modularly constructed to include any number and type of tissue models, allowing simultaneous representation of a tissue's diffusivity, orientation, volume fractions, axon orientation dispersion, and axon diameter distribution. In particular, Dmipy is geared toward facilitating reproducible, reliable MC-modeling pipelines, often allowing the whole process from model construction to parameter map recovery in fewer than 10 lines of code. To demonstrate Dmipy's ease of use and potential, we implement a wide range of well-known MC-models, including IVIM, AxCaliber, NODDI(x), Bingham-NODDI, the spherical mean-based SMT and MC-MDI, and spherical convolution-based single- and multi-tissue CSD. By allowing parameter cascading between MC-models, Dmipy also facilitates implementation of advanced approaches like CSD with voxel-varying kernels and single-shell 3-tissue CSD. By providing a well-tested, user-friendly toolbox that simplifies the interaction with the otherwise complicated field of dMRI-based Microstructure Imaging, Dmipy contributes to more reproducible, high-quality research.

## 1. Introduction

For over three decades, multi-compartment (MC) modeling has played a major role in driving diffusion MRI (dMRI)-based microstructure research. It has enabled breakthroughs in our understanding of the orientation of white matter pathways in the brain (e.g., Basser et al., 1994; Behrens et al., 2003; Tournier et al., 2007), axon bundle dispersion (e.g., Kaden et al., 2007; Zhang et al., 2012), axon diameter (e.g., Assaf et al., 2008), extra-axonal diffusivity (e.g., Novikov et al., 2018), and tumor composition (e.g., Le Bihan et al., 1988; Panagiotaki et al., 2014). Importantly, in all these works, the valid interpretation of estimated model parameters with respect to tissue composition always hinges on:

- The appropriateness of the MC-model composition in terms of *biophysical models* for the tissue of interest (Panagiotaki et al., 2012; Ferizi et al., 2016);
- The sensitivity of the dMRI acquisition to the tissue feature of interest (Ning et al., 2015);
- The specificity of the MC-model's parameters to the tissue feature of interest (Jelescu et al., 2016);
- Given an MC-model, the robustness and accuracy of the optimization approach that estimates the model parameters from the measured data (Harms et al., 2017; Canales-Rodríguez et al., 2019).

To leverage and showcase the reproducibility of published works, several platforms have released *application-specific* toolboxes for particular MC-model implementations. Well-known examples are UCL's collected works on MC-based microstructure estimation (Cook et al., 2006)^1^, MRtrix's works aimed at fiber tractography (Tournier et al., 2012)^2^, and Dipy's wide variety of dMRI-based signal models (Garyfallidis et al., 2014)^3^. While valuable, static implementations of MC-models are limited in that they cannot be easily adjusted for use case specific tissue configurations, data acquisitions, or desired optimization algorithms. On the other hand, implementing your own MC-modeling approach from scratch requires non-trivial knowledge of computer science and optimization theory, often leaving the user no choice but to use what is available.

Dmipy (*Diffusion Microstructure Imaging in Python*) is an open-source software solution based on the idea that MC-modeling—and all that involves—should be transparent, reproducible, and most of all *easy*. To this end, Dmipy facilitates the on-the-fly design, optimization and analysis of custom MC-models for any PGSE-based dMRI acquisition. Dmipy enables having only high-level interaction with model design and parameter recovery, which in turn highly simplifies the implementation of complex state-of-the-art MC-models. In fact, most MC-models in literature can be reproduced in around 10 lines of code, which we demonstrate in this work for IVIM (Le Bihan et al., 1988), ActiveAx (Alexander et al., 2010), AxCaliber (Assaf and Pasternak, 2008), Ball and Stick (Behrens et al., 2003), NODDI(-x) (Zhang et al., 2012), NODDI-Bingham (Tariq et al., 2016), MC-MDI (Kaden et al., 2016), Multi-Tissue CSD (Jeurissen et al., 2014), and Single-Shell Multi-Tissue CSD (Dhollander and Connelly, 2016).

**Figure 1:**
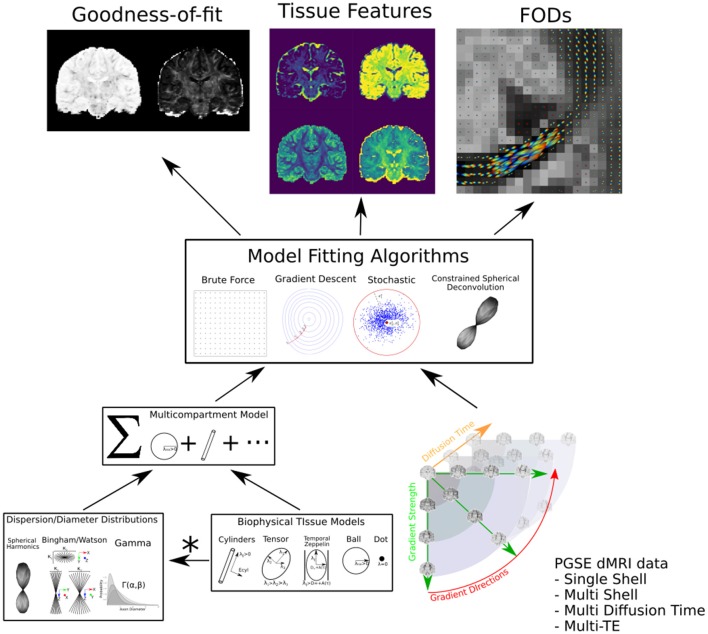
Dmipy workflow: Modular microstructure model setup and parameter estimation. Different biophysical tissue models (see Figure 3) are dispersed and/or distributed and combined together in a multi-compartment model, which is then fit to diffusion data using a chosen optimization algorithm to estimate tissue feature parameters, reconstruct FODs, and quantify the quality of the fitting.

We provide the graphical abstract Dmipy in Figure 1. Dmipy's design is based on the observation that one can view different formulations biophysical models as “building blocks,” which could be assembled in any combination, and whose meaning can change depending on the application (e.g., Panagiotaki et al., 2012; Fick, 2017). This is despite the fact that the individual tissue models, such as various approximations of restricted geometries like cylinders and spheres (e.g., Balinov et al., 1993; Vangelderen et al., 1994; Callaghan, 1995), can have quite complex mathematical formulations. To some extent, several open-source solutions have already taken advantage of this observation for applications such as modular signal generation with generalized acquisition parameters in MISST (Ianuş et al., 2016), or GPU-accelerated model generation and optimization in MDT (Harms et al., 2017).

In this work, however, we observe that this notion of modularity goes much further than previously explored, and includes alternative dMRI representation frameworks such as the multi-compartment *spherical mean* (MC-SM) models (e.g., Kaden et al., 2015) and multi-compartment *spherical harmonics* (MC-SH) models (e.g., Jeurissen et al., 2014). As a consequence, this also implies the generalization of optimization strategies that have been specifically proposed for fitting the dMRI signal, such as Microstructure Imaging in Crossings (MIX) (Farooq et al., 2016), and multi-tissue CSD (Jeurissen et al., 2014), to any MC-model composition. In particular, by generalizing MC, MC-SM, and MC-SH models to use the same biophysical models, it becomes natural to start exploring *cross-MC-modeling* approaches, where one set of parameters is estimated in one framework and then used to initiate the optimization in another framework or model composition (e.g., Nath et al., 2019; Pizzolato et al., 2018).

In Dmipy, we take this “building-block”-based philosophy on MC-modeling to the next level, and have implemented a “model-agnostic” MC-model-generation and estimation framework that can:

- Assemble any combination of biophysical models into any of these three MC-model frameworks on-the-fly;
- Add parametric and non-parametric parameter distributions to model *axon orientation dispersion* and/or *axon diameter distribution* effects for any appropriate tissue model;
- Impose any predefined or custom parameter constraints or relations;
- Optimize models using generalized, open-source implementations of various optimization strategies;
- Simulate the signal and estimate parameters from any user-defined PGSE-dMRI acquisition, varying over gradient strengths (multi-shell), diffusion times, and echo times.

Dmipy is freely available under open-source MIT license at https://github.com/AthenaEPI/dmipy, where detailed tutorials and implementations of many MC-models in literature are provided in the form of Jupyter Notebooks. As for dependencies, Dmipy's dynamic modeling design is based on numpy (Oliphant, 2006), non-linear optimization on scipy (Jones et al., 2001), convex optimization on cvxpy (Diamond and Boyd, 2016; Agrawal et al., 2018) and for signal-based subroutines (e.g., DTI) and visualization we use dipy (Garyfallidis et al., 2014). As optional dependencies, we speed up code use Numba's just-in-time compilation (Lam et al., 2015), and allow multi-core CPU processing using pathos (McKerns et al., 2012).

The structure of this paper is as follows: in section 2, we explain the theory and basic interaction behind generalizing MC-modeling over the three modeling frameworks, for any PGSE acquisition scheme and biophysical model composition. In particular, we provide Dmipy's technical aspects and workflow, explaining how the generalized signal simulation, parameter linking and optimization can be used. In section 3 we then show Dmipy in action, implementing, and demonstrating various state-of-the-art MC-modeling approaches. Finally, we discuss Dmipy's contribution to dMRI microstructure imaging and further opportunities in section 4, and provide concluding remarks in section 5.

## 2. Theory and Implementation

The goal of the Dmipy framework is to allow for the natural, generalized implementation of MC-based microstructure recovery, based on the PGSE-dMRI sequence. In this section, we explain both the theory of MC-modeling and how it is implemented in Dmipy. Note, however, that we only consider the analytic description of any algorithms we introduce, leaving the numerical implementation for the Supplementary Material. As this section describes and implements a considerably large part of the literature, we provide a table of contents in Table 1.

**Table 1:** Table of contents of sections.

**Section description**	**Section**
PGSE Diffusion Contrast	2.1
DmipyAcquisitionScheme	2.2
Multi-Compartment Modeling	2.3
Generalized 3D Signal Representations	2.4
Microscopic Compartment Models	2.5
Gaussian Compartment Models	2.5.1
Restricted CompartmentModels	2.5.2
Macroscopic Distibuted Models	2.6
Parameter Distributed Models	2.6.1
Spherical Distributed Models	2.6.2
Tissue Response Models	2.6.3
Multi-Compartment Model Variants	2.7
Multi-Compartment Spherical Mean	2.7.1
Multi-Compartment Spherical Harmonics	2.7.2
Parameter Linking	2.8
Generalized Optimization	2.9
Optimizing MC and MC-SM Models	2.9.1
Optimizing MC-SH Models	2.9.2
Fitting the Signal: Multi-tissue modeling	2.9.3

### 2.1. PGSE Diffusion Contrast

**Figure 2:**
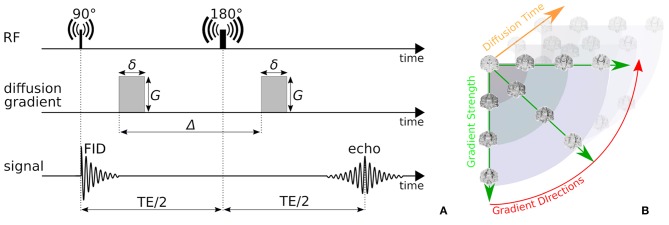
**(A)** A schematic representation of a Pulsed-Gradient Spin Echo (PGSE) sequence, and **(B)** illustrates the whole-brain diffusion contrasts in a four-dimensional, spatio-temporal PGSE acquisition, varying over gradient strength, gradient direction, and diffusion time. Notice that depending on the tissue configuration, different acquisition parameter combinations result in different image contrasts.

In this work, we focus on probing the tissue microstructure using the standard Pulsed Gradient Spin-Echo sequence (PGSE) to obtain diffusion-weighted images (DWIs) (Stejskal, 1965). We provide a schematic representation of a PGSE sequence in Figure 2A. To summarize, a diffusion-weighted measurement at position **x** ∈ ℝ^3^ is obtained by first applying a 90° radio-frequency pulse, after which two sensitizing diffusion gradients of pulse length δ [s], gradient strength *G* [T/m], and separated by separation time Δ [s], are applied to the tissue along orientation **n** ∈ 𝕊^2^. In between the two pulses, at half the Echo Time (TE), a 180° pulse is applied, resulting in a measurable spin echo signal at TE. The PGSE sequence is sensitive to diffusion in that the measured signal attenuates proportionately to the average particle motion along **n** in between the two gradient pulses. More precisely, in three dimensions, the signal attenuation for a PGSE sequence is given by $E\left(b,\text{n}\right)=\exp\left(-b{\text{n}}^{T}{\text{D}}_{\text{app}}\left(\tau \right)\text{n}\right)$, where ${\text{D}}_{\text{app}}\left(\tau \right)\in {\mathbb{R}}^{3\times 3}$ [m^2^/*s*] is the *apparent* diffusion tensor at diffusion time τ = Δ − δ/3 [s], and *b* = *G*^2^δ^2^γ^2^(Δ − δ/3) [s/m^2^] is the clinically used b-value with nuclear gyromagnetic ratio γ [s^−1^T^−1^] (Le Bihan et al., 1986; Minati and Węglarz, 2007). From these parameters, the q-value is also written as *q* = (*Gδγ*)/(2π) [m^−1^] (Callaghan, 1995).

Measuring the PGSE signal at multiple acquisition parameter combinations provides information on different tissue properties; a single-shell acquisitions (only varying **n**) allows to estimate tissue orientation (Basser et al., 1994; Tuch, 2004; Tournier et al., 2007); multi-shell (varying **n**, *G*) allows to delineate the signal contribution of multiple tissue compartments and axon orientation dispersion (Wu and Alexander, 2007; Zhang et al., 2012); multi-diffusion time (varying *G*, δ, Δ) allows to estimate e.g., axon diameter distributions (Assaf et al., 2008; De Santis et al., 2016); and multi-Echo Time (TE) is needed to recover myelin content in white matter (Whittall and MacKay, 1989). We illustrate the DWI diffusion contrast in a coronal brain slice over varying **n**, *G*, Δ in Figure 2B.

Given the PGSE acquisition parameters, the measured signal *S* is separable in terms of its non-diffusion weighted signal intensity *S*_0_ when *G* = 0, and the signal *attenuation* *E* when *G* > 0, such that

(1) $$\begin{array}{r} S\left(\text{x},G,\text{n},\Delta ,\delta ,TE\right)=S_{0}\left(\text{x},TE\right)\cdot E\left(\text{x},G,\text{n},\Delta ,\delta \right). \end{array}$$

Notice that at any position **x** the signal amplitude *S*_0_ is only dependent on *TE*, and the signal's shape *E* is dependent on all other parameters except *TE*.

### 2.2. Representing Any PGSE Acquisition Using a DmipyAcquisitionScheme

To allow Dmipy to interact with any PGSE acquisition scheme, the DmipyAcquisitionScheme module takes the raw PGSE acquisition scheme parameters and prepares them to be used for signal generation. A DmipyAcquisitionScheme can be instantiated in three ways:

- For clinical data, using b-values [*s*^2^/*m*] and gradient directions (and optionally δ/Δ/TE [*s*]);
- Using G [*T*/*m*], gradient directions, δ and Δ (and optionally TE);
- Using q [*m*^−1^], gradient directions, δ and Δ (and optionally TE).

We show how to create a scheme for a clinical acquisition in python Snippet 1. Notice that pulse duration, pulse separation, and TE can be given as a single number (it does not change throughout the acquisition), or an array the same size as b-values, potentially changing for every single measurement. Once the scheme is created, a summary can be generated indicating the total number of measurements, shells, b0-measurements, and a shell-wise description of the acquisition parameters. Measurements are automatically separated into distinct acquisition shells using a simple linkage clustering algorithm (Müllner, 2011), which clusters measurements that are closer together than a certain distance in *b*-space. Measurements having different δ/Δ/*TE* are clustered separately, ensuring measurements with similar *b*-value but different times are never combined into one shell.

**Snippet 1:**
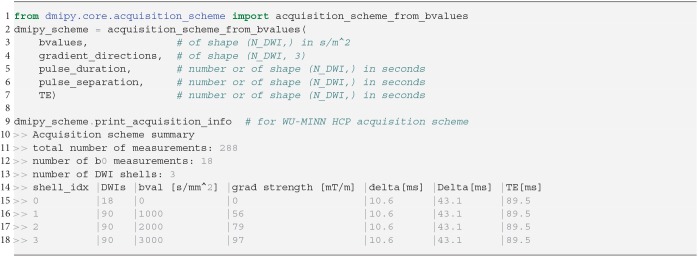
Typical DmipyAcquisitionScheme creation using b-values, gradient orientations as 3D unit vectors, and optional single-value or arrays of pulse durations δ, pulse separations Δ and TEs. The DmipyAcquisitionScheme handles all interactions with the fixed acquisition parameters, meaning we only need to focus on CompartmentModel parameters afterwards. Notice that the printed acquisition information is not in SI units to avoid too small or large numbers, but the input must always be given in SI units.

To facilitate the notation of separate DWIs per acquisition shell, let us denote the sampled acquisition scheme parameters as **A** = {[*b*_*i,s*_, *G*_*i,s*_, **n**_*i,s*_, δ_*i,s*_, Δ_*i,s*_, *TE*_*i,s*_]}. Here, we used double linear indexing *i,s*, such that *s* = 1, 2, …, *N*_shells_ is the acquisition shell index with *N*_shells_ the number of shells, and *i* = 1, 2, …, *N*_DWI_[*s*] is the DWI index with *N*_DWI_[*s*] the number of DWIs for shell *s*. We will also use a *shell-wise* acquisition scheme **A**_*s*_ = {[*b*_*s*_, *G*_*s*_, δ_*s*_, Δ_*s*_, *TE*_*s*_]} that omits the gradient directions of that shell **n**_*s*_, and only contains the unique values of the other parameters for each shell.

### 2.3. Anatomy of dMRI Multi-Compartment Modeling

To recover information on the tissue microstructure, the attenuation of the signal must first be explained in terms of the underlying diffusion process—the Ensemble Average Propagator (EAP). Using the *short gradient pulse* approximation (SGP), i.e., assuming that no diffusion takes place during the gradient pulse (δ → 0), the relation between the signal attenuation and the EAP is given by a Fourier transform (Stejskal, 1965). This transforms Equation (1) into

(2) $$\begin{array}{r} E\left(\text{x},\text{A}\right)\overset{\text{SGP}}{=}\int_{{\mathbb{R}}^{3}}P\left(\text{x},\text{R};\tau \right)e^{\left(-i\gamma \delta \text{G}\right)\cdot \text{R}}d\text{R} \end{array}$$

where *P*(**x**, **R**; τ) describes the probability that a particle anywhere inside voxel **x** traveled a net distance *R* = ||**R**|| [m] *given* a diffusion time τ. Here, **R** = *R***r** where **r** ∈ 𝕊^2^ and non-negative *R* ∈ ℝ^+^. Hypothetically, given sufficiently small resolution and dense measurements in *G*, **n**, τ, one can directly recover and estimate microstructure-related properties of the EAP for a single τ using an inverse Fourier transform or Fourier basis (e.g., Wedeen et al., 2005; Özarslan et al., 2013; Fick et al., 2016), or over continuous τ (Fick et al., 2018). However, such approaches still hinge on the appropriateness of the SGP approximation, and can only describe properties of the overall signal and EAP, not of specific tissues in heterogeneous environments.

To estimate specific properties of the tissue microstructure, Multi-Compartment (MC)-models represent the tissue as a linear combination of single compartment models “*C*,” each representing the diffusion signal originating from specific tissue types. Recovering tissue microstructure information is done by finding the model parameters **p** that minimize the difference between modeled and the measured signal. The process of MC-modeling any model containing *i* ≥ 1 compartments is thus given by

(3) $$\begin{array}{r} {\text{p}}^{*}\left(\text{x}\right)={\text{argmin}}_{\text{p}}\int {\left[E\left(\text{x},\text{A}\right)-{\hat{E}}^{\text{MC}}\left(\text{A},\text{p}\right)\right]}^{2}d\text{A}, \end{array}$$

(4) $$\begin{array}{r} \text{with }{\hat{E}}^{\text{MC}}\left(\text{A},\text{p}\right)=\overset{N}{\underset{i}{\sum }}f_{i}C_{i}\left(\text{A},{\text{p}}_{i}\right) \end{array}$$

where the parameters of each model **p**_*i*_ represent its tissue microstructure properties like diffusivity, orientation, axon diameter, dispersion, and others. The non-negative, normalized volume fractions *f*_*i*_ weight the signal contribution of each compartment model. For these parameters to accurately describe the underlying tissue configuration, it is essential that the MC-model composition in terms of compartment models is appropriate for the actual tissue composition. Notice that Equation (3) is general, and its compartments do not necessarily need to be based on the SGP condition in Equation (2). In the next sections, we show which compartment model representations are available in Dmipy, and how they can be combined into an MC-model and be prepared for optimization.

### 2.4. Dmipy's Generalized Three-Dimensional Signal Representations

**Figure 3:**
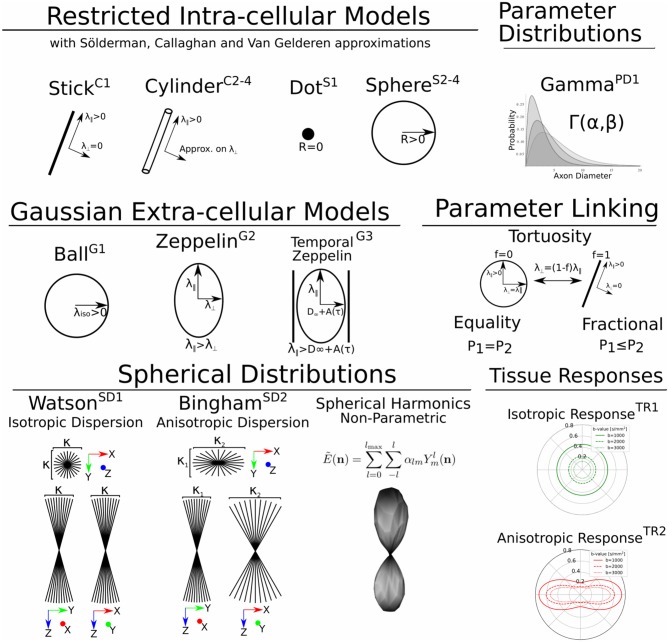
A schematic of most biophysical mode ls that are used in PGSE-based Microstructure Imaging. Using different combinations of these “components,” any microstructure model can be assembled using Dmipy.

In Dmipy, the single models *C*(**A**, **p**) that make up the MC-model in Equation (3) are represented using generalized three-dimensional signal representations, called “CompartmentModels” or “DistributedModels.” The CompartmentModel is the basic block that contains the signal representation of any single, undistributed biophysical model. CompartmentModels are either isotropic or anisotropic, but axially symmetric. Anisotropic models have an orientation **μ** ∈ 𝕊^2^ and are described as a separable product of the one- and two-dimensional models, representing their parallel and perpendicular components, respectively (Assaf et al., 2004). DistributedModels consist of other CompartmentModels or DistributedModels and apply parametric distributions on the parameters of its input models. We illustrate Dmipy's available model representations in Figure 3 with their associated parameters. By combining these models in various combinations, it is possible to construct nearly any MC-model in the literature. In Table 2 we explicitly write out the unique parameters of these models, provide their cardinality, SI unit, and in which models they are present.

**Table 2:** Overview of CompartmentModel parameters with their associated cardinality, SI-units, in which models they occur and description.

**Parameter name**	**Card**.	**SI unit**	**Present in models**	**Description**
lambda_iso	1	*m*^2^/*s*	G1	Isotropic Gaussian diffusivity
lambda_par	1	*m*^2^/*s*	G2-3, C1-4	Parallel Gaussian diffusivity
lambda_perp	1	*m*^2^/*s*	G2	Perpendicular diffusivity
lambda_inf	1	*m*^2^/*s*	G3	Bulk diffusivity
lambda_intra	1	*m*^2^/*s*	C3-4, S3-4	Intra-cellular diffusivity
A	1	*m*^−2^	G3	Characteristic coefficient
mu	2	[rad]	G2-3, C1-4, SD1-2, TR2	Orientation θ/ϕ Euler angles
diameter	1	*m*	C2-4, S2-4	Cylinder/Sphere diameter
odi	1	[-]	SD1-2	Orientation Dispersion Index
beta	1	[-]	SD2	Secondary Bingham dispersion
alpha, beta	1	[-]	PD1	Shape/Scale of Gamma distribution
sh_coeff	1_max_	[-]	MC-SH	FOD SH-coefficients
partial_volume_n	1	[-]	MC, MC-SM, MC-SH	volume fraction

To generate the signal representation for any input model *C*(**A**, **p**)—regardless of the mathematical representation—we only need values for its parameters **p** and the acquisition parameters **A**, represented in a DmipyAcquisitionScheme. Having this input, a CompartmentModel or DistributedModel can return three main signal *attenuation* representations to be used by the higher-level multi-compartment and distributed models:

- A standard representation $\hat{E}\left(\text{A}\right)=C\left(\text{A},\text{p}\right)\in {\mathbb{R}}^{N_{\text{DWI}}}$, evaluating the biophysical model for the given acquisition parameters $\tilde{\text{A}}$, where *N*_DWI_ is the number of acquired DWIs.
- A spherical mean representation ${\hat{E}}_{SM}\left({\text{A}}_{s}\right)=\frac{1}{4\pi }\int_{{𝕊}^{2}}C\left({\text{A}}_{s},\text{n};\text{p}\right)d\text{n}\in {\mathbb{R}}^{N_{\text{shells}}}$, returning the shell-wise spherical mean of the signal. If no analytic expression is available, then it is estimated numerically.
- And a multi-shell convolution kernel matrix $\text{M}\left({\text{A}}_{s},\text{p}\right)\in {\mathbb{R}}^{N_{\text{DWIs}}\times N_{\text{SH-coef}}}$, which enables convolution of the model's rotational harmonics representation with the spherical harmonics expansion of some spherical distributions with number of coefficients *N*_SH-coef_. See details in the Supplementary Material.

In Snippet 2, we provide the code to instantiate all the available microscopic CompartmentModels that we will present in the next section 2.5. The signal generation for the Distributed Models we present in section 2.6 is analogous.

**Snippet 2:**
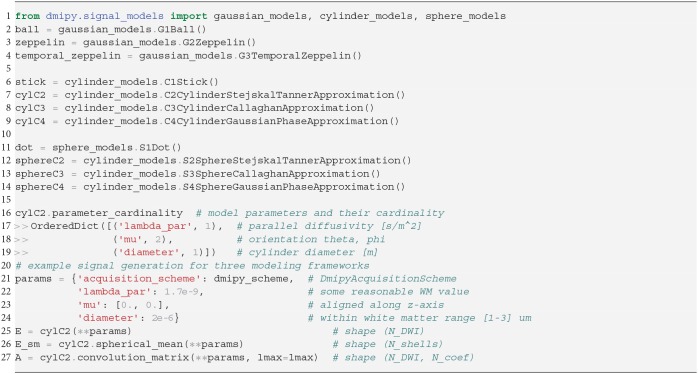
One-line instantiation of available CompartmentModel representations in Dmipy. For any of the models the parameter cardinality and names can be inspected. Signal representations for any DmipyAcquisitionScheme can be generated by providing valid values for the parameters.

### 2.5. Microscopic Compartment Models

Parsimonious models of the tissue microstructure are usually based on what we know the tissue looks like from histological observations. Well-known examples are using cylinders of some diameter to represent axons (Aboitiz et al., 1992; Assaf et al., 2008), or spheres of some diameter to represent tumor cells (Panagiotaki et al., 2014). In this section, we describe the way Dmipy allows modular signal generation for any microscopic model, and which models are available. These include Gaussian models (G) in section 2.5.1, restricted models such as cylinders (C) and spheres (S) in section 2.5.2.

#### 2.5.1. Gaussian Models (G)

In MC-modeling, Gaussian models are used to represent free or hindered diffusion inside tissues that are not explicitly restricting the movement of diffusing particles. Essentially, they say a particle could travel anywhere in **R** ∈ ℝ^3^ with non-zero, but exponentially decreasing probability as *R* → ∞. They are convenient to represent the Fourier-like relationship between the signal and EAP, as the closed-form Fourier transform of a Gaussian is another Gaussian.

**G1**: The simplest Gaussian model is the Ball: an isotropic Gaussian compartment whose signal attenuation only depends on isotropic diffusivity λ_iso_[m^2^/s]. Balls usually represent the diffusion signal of free water (Behrens et al., 2003), or tissue types that on average induce an isotropic hindrance on the EAP, like gray matter (Dell'Acqua et al., 2010).

**G2**: The Zeppelin is an axially symmetric Gaussian distribution aligned along orientation **μ**, with parallel and perpendicular diffusivity λ_∥_ ≥ λ_⊥_[m^2^/s]. It is often used to describe the diffusion signal originating from the oriented, extra-axonal space (Alexander et al., 2010; Zhang et al., 2012).

**G3**: To represent extra-axonal diffusion, recent works argue that hindered diffusion is actually slower-than-exponential over τ due to how the external axon boundaries still restrict diffusing particles (Novikov et al., 2014). To account for this, Burcaw et al. (2015) proposed a modification to the Zeppelin we refer to as the Temporal Zeppelin, where the perpendicular diffusivity is instead given by $\lambda_{⊥}=\lambda_{\infty }+\frac{A\ln\left(\Delta /\delta \right)+3/2}{\Delta -\delta /3}$, with λ_∞_[m^2^/s] the bulk diffusivity for large diffusion times, and *A*[m^−2^] the characteristic coefficient for the packing of the axons. The Temporal Zeppelin has been used by De Santis et al. (2016) to characterize extra-axonal hindrance in tandem with axon diameter estimation.

#### 2.5.2. Restricted Cylinder (C) and Sphere (S) Models

Diffusion *restriction* is the phenomenon of particles diffusing during pulse separation time Δ, exploring the geometry and finding they cannot move through boundaries at a certain distance *D*[m] from their origin point, i.e., *P*(**R**) > 0, ∀||**R**|| ≤ *D*. The relationship between the EAP and the diffusion signal inside Cylinders (C) and Spheres (S) has been well studied (Stejskal, 1965; Balinov et al., 1993; Vangelderen et al., 1994; Callaghan, 1995). The most commonly used applications of these models are Cylinders to represent axons (Assaf et al., 2004), and Spheres to represent cell bodies (Panagiotaki et al., 2014), such that the diameter of the cylinder or sphere represents the diameter of axons and cells. Sphere models are isotropic with a diameter *D* and intra-spherical diffusivity λ_intra_. Cylinder models are formulated as anisotropic models, having Gaussian diffusivity λ_∥_ along orientation **μ**, and Cylinder diameter *D* and intra-cylindrical diffusivity λ_intra_ perpendicular to it.

**C1, S1**: Both the Cylinder and Sphere models have implementations that assume the geometry diameter is negligible and can be set to zero. This results in the well-known Stick model (C1) (Behrens et al., 2003) and non-diffusing Dot model (S1) (Panagiotaki et al., 2012).

**C2-4, S2-4**: To relate the EAP to the measured signal for restricted models, several assumptions on the diffusion process are used. Dmipy implements three different approximations of Cylinders and Spheres, which we order by decreasingly stringent assumptions on the diffusion process. The most stringent is the “Stejskal-Tanner” approximation (C2, S2), which simultaneously assumes the previously described SGP, and the long diffusion time limit. The “Callaghan” approximation allows finite diffusion time, only assuming the SGP (C3, S3). Finally, the “Gaussian Phase” approximation only assumes the diffusion is Gaussian during pulse length δ (C4, S4). We note that C4, S4 are examples of models that are not based on the SGP condition in Equation (2). Details can be found in the Supplementary Material.

### 2.6. Macroscopic Distributed Models

DistributedModels include features that are properties of groups of microstructural features, like the distributions of axon diameter (Assaf et al., 2008) and axon orientation (Kaden et al., 2007; Tournier et al., 2007) inside the same white matter bundle. There are two types of DistributedModels in Dmipy. The first is the “ParameterDistributed” (PD) model for one-dimensional distributions over e.g., axon diameter, which we describe in section 2.6.1. The second is the “SphericalDistributed” (SD) model for parametric distributions of models on the sphere for e.g., axon dispersion (Leergaard et al., 2010) in section 2.6.2. We also describe “TissueResponse” models, which are non-parametric representations of the macroscopic measured signal, in section 2.6.3.

#### 2.6.1. Parametric Distributions Over Model Parameters (PD)

A parameter distributed (PD) model can apply parametric distributions over parameters of cardinality one, like diffusivity or cylinder diameter (see Table 2). We define a parametric distribution as *P*(ξ; **Ω**) with **Ω** ∈ ℝ its parameters and ξ ∈ ℝ^+^ the parameter that is sampled. We can then define any parameter distributed compartment model *C*_*PD*_ as

(5) $$\begin{array}{r} C_{PD}\left(\text{A};\tilde{\text{p}},\Omega \right)=\frac{\int_{{\mathbb{R}}^{+}}\overset{\text{PDF}}{\overset{︷}{P\left(\xi ;\Omega \right)}}\cdot \overset{\text{(Restricted) Signal Attenuation}}{\overset{︷}{C\left(\text{A};\xi ,\tilde{\text{p}}\right)}}\cdot \overset{\text{Volume Correction}}{\overset{︷}{N\left(\xi \right)}}d\xi }{\underset{\text{Normalization}}{\underset{︸}{\int_{{\mathbb{R}}^{+}}P\left(\xi ;\Omega \right)\cdot N\left(\xi \right)d\xi }}} \end{array}$$

where $\left\{\xi ,\tilde{\text{p}}\right\}\in \text{p}$ together are all parameters of model *C*. The distributed parameter ξ is integrated out, and replaced by the distribution parameters **Ω**. When distributing diffusivity the normalization is *N*(ξ) = 1, but, in the case of distributing the radii of restricted models such as planes, cylinders, or spheres, *N*(ξ) is the volume function of that geometry. Briefly, *N*_plate_(ξ) = ξ, $N_{\text{cylinder}}\left(\xi \right)=\pi \xi^{2}$ and $N_{\text{sphere}}\left(\xi \right)=\frac{4}{3}\pi \xi^{3}$. The reason for this is that it is not the geometries themselves, but the simulated particles diffusing inside these geometries that are contributing to the signal attenuation. In Snippet 3, line 9 it is demonstrated that any model parameter can be distributed only by providing its name upon initialization of the PD model.

**Snippet 3:**
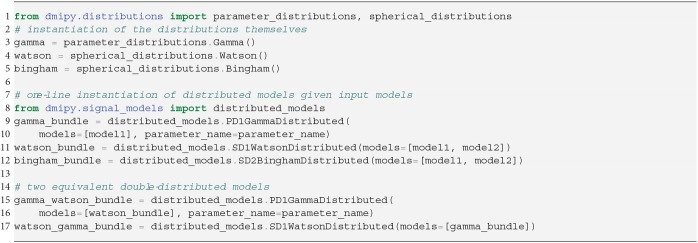
One-line instantiation of parameter and spherical distributions and DistributedModels. Notice DistributedModels can be seen as sub-MC-models that apply a distribution to the parameters of base CompartmentModels.

**PD1**: A Gamma distribution with shape parameter α and scale parameter β has been used to model the axon diameter distribution in nervous tissue (Aboitiz et al., 1992; Assaf et al., 2008).

#### 2.6.2. Spherical Distributions Over Model Orientation (SD)

Spherical distributed (SD) models apply parametric distributions of over model orientation. The signal attenuation of a SD model is obtained by means of a spherical convolution of spherical distribution F(**n**; **Ω**):𝕊^2^ → [0, ∞] with parameters **Ω**, with one or more convolution kernel models $C_{i}^{K}\left(\text{n},{\tilde{\text{p}}}_{i}\right)=C\left(\text{n},{\tilde{\text{p}}}_{i}|\mu_{i}=\left[0,0,1\right]\right)$, meaning ${\tilde{\text{p}}}_{i},\mu_{i}\in {\text{p}}_{i}$ and the orientation **μ**_*i*_ is parallel to the z-axis. An SD model is thus given by

(6) $$\begin{array}{r} C_{\text{SD}}\left({\text{A}}_{s},\text{n};\left\{{\tilde{\text{p}}}_{i}\right\},\Omega \right)=\int_{{𝕊}^{2}}F\left(\text{n}-\text{u};\Omega \right)\cdot \overset{N}{\underset{i=1}{\sum }}f_{i}C_{i}^{K}\left({\text{A}}_{s},\text{u};{\tilde{\text{p}}}_{i}\right)d\text{u} \end{array}$$

(7) $$\begin{array}{r} \;=\left[F\left(\Omega \right){*}_{{𝕊}^{2}}\overset{N}{\underset{i=1}{\sum }}f_{i}C_{i}^{K}\left({\text{A}}_{s};{\tilde{\text{p}}}_{i}\right)\right]\left(\text{n}\right), \end{array}$$

where *f*_*i*_ are the normalized volume fractions weighting the signal contribution of each convolution kernel, **A**_*s*_ the shell-wise acquisition parameters and **u** ∈ 𝕊^2^ the integrated orientation variable. Notice that the spherical convolution is done separately for each shell *s*. We provide details on the numerical implementation of Equation (6) in the Supplementary Material. We will use the second shorthand in Equation (7) for spherical convolution hereafter.

SD-models can be seen as a sub-MC-model, which can, in turn, be combined with other compartment models in Equation (3). In Snippet 3, lines 10 and 11, we illustrate how an SD model is instantiated given one or more input models. Note that, in this way, *multiple* convolution kernels are distributed by *the same* spherical distribution. As in PD models, in SD models the orientation parameters of the input models are removed, and the parameters of the spherical distribution are added. Note that we can stack and apply multiple distributions to parameters of input models. As a concrete example, using lines 15 and 17, we can simulate the signal from a white matter bundle whose axons are simultaneously distributed over diameter and dispersed over orientation.

**SD1**: The Watson distribution, defined as an isotropic Gaussian distribution on the sphere with orientation **μ** and concentration parameter κ. Following Zhang et al. (2012), we use the orientation dispersion index (ODI) as an optimization parameter, defining *ODI* = 0 as a spike function along **μ**, and *ODI* = 1 as an isotropically dispersed profile on the sphere.

**SD2** The Bingham Distribution, defined as an anisotropic Gaussian distribution on the sphere with orientation **μ** and primary and secondary concentration concentrations κ_1_, κ_2_. Following Tariq et al. (2016), analogously to SD1, we use optimization parameters *ODI* and β in lieu of the concentrations. The Bingham has an additional ψ “roll” parameter, which rotates the distribution about **μ**.

#### 2.6.3. Tissue Response Models (TR)

Tissue Response (TR) models are non-parametric representations of the measured signal, which are recovered by averaging the signal in segmented voxels of specific tissue types. These models differ from the previously described parametric models in that they are not related to the “true” underlying EAP of the tissue. In Snippet 4 we provide the code to produce isotropic (TR1) and anisotropic (TR2) tissue response models, given some data segmentation. Formally, let **X**_iso_ and **X**_aniso_ be segmented voxels of isotropic and anisotropic diffusion profiles. Tissue response models are then created by first representing the signal in each voxel as rotational harmonics coefficients **r** with order *l* using a rotational harmonics transform (RHT)—see the Supplementary Material—and then averaging those coefficients. In doing so, we can separate the segmented tissue-specific *S*_0_ signal response and its shell-wise shape, allowing for so-called multi-tissue modeling (Jeurissen et al., 2014).

**Snippet 4:**
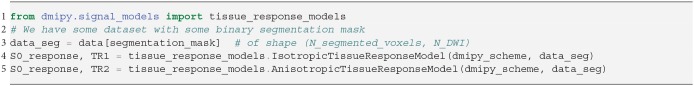
Instantiation of anisotropic and isotropic tissue response models, given some data segmentation. Notice that we can produce an isotropic or anisotropic TR-model regardless of the data, and that we also recover the mean signal amplitude *S*_0_ of the input data, which we can use later when setting up MC-models in Snippet 5.

**TR1**: The isotropic tissue response model is given by

(8) $$\begin{array}{l} S_{0}C_{\text{TR1}}\left({\text{A}}_{s},{\text{r}}_{s,l=0}\right)=\frac{1}{N_{\text{vox}}}\overset{N_{\text{vox}}}{\underset{i=1}{\sum }}\text{RHT}\left[S\left({\text{x}}_{i},{\text{A}}_{s}\right)\right]\;\text{for}\\ \;{\text{x}}_{i}\in {\text{X}}_{\text{iso}}. \end{array}$$

Notice that we recover the tissue-specific baseline intensity *S*_0, iso_, and that only the *l* = 0 coefficients are needed to represent isotropic profiles.

**TR2**: The anisotropic tissue response model is given as

(9) $$\begin{array}{l} S_{0}C_{\text{TR2}}\left({\text{A}}_{s},{\text{r}}_{s,l},\mu \right)=\frac{1}{N_{\text{vox}}}\overset{N_{\text{vox}}}{\underset{i=1}{\sum }}\text{RHT}\left[S\left({\text{x}}_{i},{\text{R}}_{i}{\text{A}}_{s}\right)\right]\;\text{for}\\ \;{\text{x}}_{i}\in {\text{X}}_{\text{aniso}}. \end{array}$$

where we note that the TR2 model has an orientation **μ** and gradient orientations **n**_*s*_ are rotated with rotation matrix **R** such that the signal orientations (according to a DTI tensor) is pointing along the z-axis.

### 2.7. Multi-Compartment Modeling Variants

In section 2.3 we introduced the definition of standard MC-modeling. Dmipy, however, also implements alternative MC-modeling frameworks. In this section, we introduce the Multi-Compartment Spherical Mean (MC-SM) variant in section 2.7.1 and the Multi-Compartment Spherical Harmonics (MC-SH) variant in section 2.7.2.

#### 2.7.1. Multi-Compartment Spherical Mean Model (MC-SM)

To estimate properties of the tissue microstructure that are independent of axon orientation dispersion and crossings, Kaden et al. (2015) noticed that the shell-wise spherical mean of the measured signal is invariant to these effects. Taking the spherical convolution in Equation (7), it is straightforward to show that the spherical mean of a spherical convolved convolution kernel is equal to the spherical mean of the kernel itself, by taking advantage of the fact that $\int_{{𝕊}^{2}}F\left(\text{n};\Omega \right)d\text{n}\equiv 1$. Recalling section 2.4, we can simply use the spherical mean representation of a compartment model in Equation (3) to estimate the parameters for the generalized Multi-Compartment Spherical Mean (MC-SM) model as

(10) $$\begin{array}{r} {\tilde{\text{p}}}^{*}\left(\text{x}\right)={\text{argmin}}_{\tilde{\text{p}}}\int {\left[E^{\text{SM}}\left(\text{x},{\text{A}}_{s}\right)-{\hat{E}}^{\text{MC-SM}}\left({\text{A}}_{s},\tilde{\text{p}}\right)\right]}^{2}d{\text{A}}_{s}, \end{array}$$

(11) $$\begin{array}{r} \;\text{with }{\hat{E}}^{\text{MC-SM}}\left({\text{A}}_{s},\tilde{\text{p}}\right)=\overset{N}{\underset{i}{\sum }}f_{i}C_{i}^{\text{SM}}\left({\text{A}}_{s},{\tilde{\text{p}}}_{i}\right), \end{array}$$

where $E^{\text{SM}}\left(\text{x},{\text{A}}_{s}\right)$ is the spherical mean of the measured signal, defined on shell-wise acquisition scheme **A**_*s*_. Here $\tilde{\text{p}}$ is again any model's parameters except its orientation **μ**, which is no longer relevant after taking the spherical mean. Using this MC-SM representation, any “standard” MC-model can be framed as a spherical mean model, enabling the creation of known models such as MC-MDI (Kaden et al., 2016), or allowing exploration of new effects like an MC-SM version of ActiveAx (Pizzolato et al., 2018).

#### 2.7.2. Multi-Compartment Spherical Harmonics Model (MC-SH)

Using spherical harmonics (SH), it is possible to represent a spherical distribution of models non-parametrically if we know beforehand what are the convolution kernels for the included tissue types. Such an MC-SH model can again be written as a generalization of Equation (3) such that

(12) $$\begin{array}{l} {\text{c}}^{*}\left(\text{x}\right)={\text{argmin}}_{\text{c}}\int {\left[E\left(\text{x},{\text{A}}_{s}\right)-{\hat{E}}^{\text{MC-SH}}\left(\text{A},\text{c}|\tilde{\text{p}}\right)\right]}^{2}d\text{A}\\ \;\text{s.t.}F_{i}\left(\text{n};\text{c}\right)\geq 0, \end{array}$$

(13) $$\begin{array}{r} \;\text{with }{\hat{E}}^{\text{MC-SH}}\left({\text{A}}_{s},\text{c}|\tilde{\text{p}}\right)=\left[\overset{N}{\underset{i=1}{\sum }}F_{i}\left({\text{c}}_{i}\right){*}_{{𝕊}^{2}}C_{i}^{K}\left({\text{A}}_{s}|{\tilde{\text{p}}}_{i}\right)\right]\left(\text{n}\right), \end{array}$$

where F(**n**;**c**) : 𝕊^2^ → [−∞, ∞] represents a non-parametric spherical distribution given in SH coefficients **c**, and where $\left(\cdot |\tilde{\text{p}}\right)$ means that kernel parameters $\tilde{\text{p}}$ are known and fixed. Notice that we must impose F(**n**; **c**) ≥ 0 as the SH distribution is not positive definite by nature. In MC-SH models, the volume fractions *f*_*i*_ are included in **c**_*i*_, and a unity constraint can be imposed if required. Note, however, that for practical reasons only one anisotropic convolution kernel may be present in Equation (13), unless their volume fractions *f*_*i*_ are fixed (as in Snippet 12).

#### 2.7.3. MC-Model Initialization

We instantiate all three MC-models in Snippet 5. Let model_1 have one orientation and one other parameter with cardinality 1, and model_2 be isotropic with only one parameter. Inspecting the MC-model parameters, notice that they are simply an aggregation of the input parameters with the addition of volume fractions, and the inclusion of S0-responses is handled internally. To avoid duplicate parameter names, each parameter name is prepended with the input model name and its enumeration. The MC-SM model is only different from MC in that the orientation parameter of model_1 is removed, as orientations have no meaning in the spherical mean signal. For the MC-SH model, the orientation is removed as the input models are used as convolution kernels, and the SH-coefficients for the given spherical harmonics order are added as parameters. To simulate the signal for a single voxel the procedure is the same as for the CompartmentModel in Snippet 2. Simulating the signal for multiple voxels is a generalization of the single-voxel simulation, which can be handled by the mc_model.simulate_signal function.

**Snippet 5:**
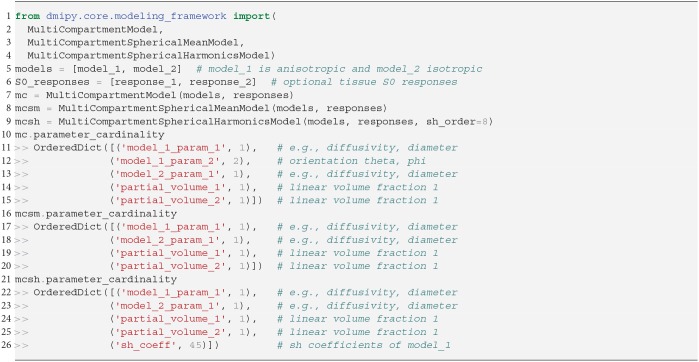
One-line instantiation of MultiCompartmentModel variants.

### 2.8. Parameter Fixing, Linking, and Optimization Parameters

After an MC-model is instantiated, the last step before the potential model fitting is the imposition of linked or fixed parameters or the addition of optimization parameters:

- Parameter linking is used to impose certain mathematical relationships between otherwise independent parameters. Examples are setting parameters equal to each other (e.g., telling two models to have the same orientation), imposing a tortuosity constraint (e.g., Szafer et al., 1995), or imposing that a parameter must be smaller or equal to another parameter (e.g., λ_⊥_ ≤ λ_∥_ for a Zeppelin);
- Parameter fixing is used to enable fixing of known global diffusion properties (e.g., CSF diffusivity), or to do parameter cascading, which is to fix voxel-wise varying parameters for a certain dataset, which have been optimized from a previous model;
- Optimization parameters are used to rescale a model parameter to a rescaled version that is more appropriate to the optimization problem, like using the Orientation Dispersion Index (ODI) in lieu of concentration parameter for a Watson distribution (Zhang et al., 2012).

In Snippet 6 we provide an example of how fixed and linked parameters can be included in instantiated distributed models and MC-models. We note that adding a tortuosity constraint can only be done at the distributed model level, and not the MC-modeling level because tortuosity turns the affected volume fraction into a non-linear parameter. At the MC-modeling level, we enforce that all volume fractions remain linear and independent, which is needed for some optimization algorithms like MIX (Farooq et al., 2016) or AMICO (Daducci et al., 2015).

**Snippet 6:**
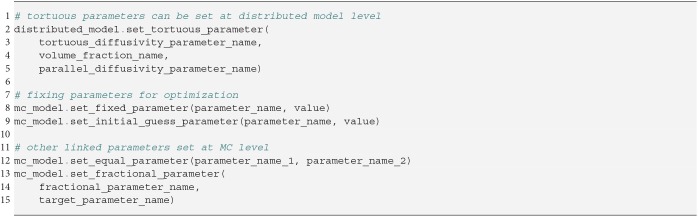
One-liners to setting fixed or linked parameters to instantiated distributed model or MC-models.

### 2.9. Generalized Optimization Strategies

Dmipy's generalized modeling approach also extends to implementations of standard optimization algorithms. We describe the fitting of MC and MC-SM models in section 2.9.1, and the fitting of MC-SH models in section 2.9.2. Finally, we discuss the secondary fitting of the signal (not the signal attenuation) for any MC-model variant in section 2.9.3.

Regardless of the MC-model variant, choosing the optimization algorithm is straightforward, as we show in Snippet 7. It suffices to just set the optimization algorithm and potentially adjust the algorithm's parameters before fitting the data. Notice that only at the fit command does the acquisition scheme come into play—before this point the model is data independent. Fitting the data returns a fitted model representation, from which the fitted parameters can be recovered.

**Snippet 7:**
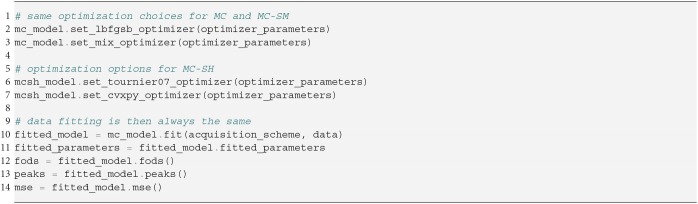
Generalized Parameter Optimization.

#### 2.9.1. Fitting MC and MC-SM Models

From the perspective of optimization, MC and MC-SM models, given in Equations (3) and (10), can be treated equally as non-convex optimization problems. Currently, we implement two optimization algorithms:

**Brute2Fine**: The standard “brute2fine” optimizer first uses brute force optimization by sampling all optimized parameters **p** on an equispaced grid of *Ns* samples between each parameter's minimum and maximum optimization bounds. Obtaining the best fitting starting position based on Equations (3) or (10), we then use the constrained optimization algorithms L-BFGS-B (Byrd et al., 1995) of the open-source scipy package (Jones et al., 2001) to obtain the locally best fitting parameters p^*^. The unity constraint on *f*_*i*_ is enforced by nesting volume fraction following Zhang et al. (2012).

**MIX**: The recent Microstructure in Crossings (MIX) algorithm (Farooq et al., 2016) is specially designed to return accurate results for highly complicated MC-models. Instead of using brute force, it uses a trick to separate the linear from non-linear parameters and uses differential evolution (Storn and Price, 1997) to find the globally best-fitting parameters. It is accurate at the cost of being slow, so we recommend “brute2fine” for simpler models.

#### 2.9.2. MC-SH Fitting

Fitting MC-SH models is different from MC or MC-SM models in that the problem can be cast in a convex optimization problem. In literature, the spherical harmonics distribution is often estimated using methods like Constrained Spherical Deconvolution (CSD) (Tournier et al., 2007; Jeurissen et al., 2014), although many variations of this approach have been proposed (Canales-Rodríguez et al., 2019). Currently, we have implemented two algorithms:

**Tournier07**: This is the classical optimization algorithm for CSD following Tournier et al. (2007). It uses the Tikhonov regularization term to penalize negative F(**n**; **c**), but not explicitly forbid it. It is a fast implementation only based on Numpy (Oliphant, 2006) but cannot handle multiple kernels, unless the volume fractions are fixed.

**CVXPY**: This implements the explicit multi-compartment CSD algorithm following Jeurissen et al. (2014) using the CVXPY package (Diamond and Boyd, 2016; Agrawal et al., 2018). It features a hard non-negativity constraint on the FOD, and can optionally enforce a unity constraint on the volume fractions. We note that enforcing the unity constraint is not recommended by Dell'Acqua and Tournier (2018) when tissue *S*_0_ responses are given.

#### 2.9.3. Secondary Multi-Tissue Optimization: Fitting the Signal

So far, we have been considering fitting the signal attenuation only, ignoring the information that is present in the signal amplitude (see Equation 1). Recent works (Jeurissen et al., 2014) show that including knowledge of the tissue-specific *S*_0_ response, and fitting the signal instead of the signal attenuation, can provide a better estimate of the linear volume fractions. We observe that this so-called Multi-Tissue modeling can be seen as a secondary convex optimization to correct the volume fractions after all non-linear parameters have been estimated. The optimization is given

(14) $$\begin{array}{r} {\text{f}}^{*}\left(\text{x}\right)=\;{\text{argmin}}_{\text{f}}\int {\left[S\left(\text{x},\text{A}\right)-\overset{N}{\underset{i}{\sum }}S_{0,i}f_{i}C_{i}\left(\text{A}|{\text{p}}_{i}^{*}\right)\right]}^{2}d\text{A}, \end{array}$$

(15) $$\begin{array}{r} \;={\text{argmin}}_{\text{f}}∥S\left(\text{x},\text{A}\right)-\text{B}\text{f}{∥}^{2} \end{array}$$

with $\text{B}=\left[S_{0,i}C_{i}\left(\text{A}|{\text{p}}_{i}^{*}\right)\right]\in {\mathbb{R}}^{N_{DWI}\times N_{models}}$ the model-wise evaluated parts of the MC-models at **A**, multiplied by the tissue-specific *S*_0_ intensity. Signal attenuation-based (shape only) MC-modeling—as we've been doing so far—implicitly assumes equal *S*_0, *i*_ = *S*_0_. This means that MC-models that model tissues with different *S*_0_ (e.g., CSF, white and gray matter) will always have biased volume fractions unless these effects are taken into account. In Dmipy, any MC-model can be corrected for this, as we showed in Snippet 5.

### 2.10. Data Set Descriptions

#### 2.10.1. IVIM Data

For our first demonstration, we will use a freely available dataset with an IVIM acquisition, which is downloadable from the dipy examples page (Garyfallidis et al., 2014). It is a small dataset with only 21 DWIs at b-values spread between 0 − 1, 000*s*/*mm*^2^, including a single *b*_0_ measurement. Information about diffusion times and TE are unknown for this data, but these will not be needed to fit the 2-compartment Gaussian IVIM model.

#### 2.10.2. Spatio-Temporal Cat Spinal Cord Data

To illustrate MC-models that are aimed at estimating axon diameter, we use a recent dataset where both AxCaliber and multi-shell diffusion MRI acquisitions have been registered to one axial slice of cat spinal cord (Duval et al., 2016). For each voxel, the mean axon diameter, restricted volume fraction and myelin volume fraction are known from histological measurements. The dMRI data is 64 × 64 voxels with resolution 0.16 × 0.16 × 0.16mm^3^. One AxCaliber acquisition was acquired (perpendicular to the axon axis) with parameters δ=3/8/8/8 ms, Δ = 7/12/25/40 ms, G = [0… 849] mT/m (199 increments) and TE minimized (36–62 ms). The data was TE-normalized by dividing the data for every TE by the G = 0 signal. The multi-shell acquisition was acquired with parameters δ = 3 ms, Δ = 30 ms, 4 shells with *b*-value={40, 189, 1,680, 6,720} s/mm^2^, TE = 47 ms, with a total of 796 diffusion-weighted images.

#### 2.10.3. Multi-Shell WU-Minn Human Connectome Project Data

To illustrate MC-models that require only multi-shell data, we use the WU-Minn Human Connectome Project data (Feinberg et al., 2010; Moeller et al., 2010; Setsompop et al., 2012; Xu et al., 2012; Glasser et al., 2013; Van Essen et al., 2013). In this dataset, the diffusion directions were obtained such that every subset of the first M directions is still isotropic (Caruyer et al., 2013). The data was sampled on 3 shells with *b*-values {0, 1,000, 2,000, 3,000}s/mm^2^, with {14, 90, 90, 90} directions, respectively. The diffusion time and pulse separation time in this data are δ/Δ = 10.6/43.1ms with 2 × 2 × 2mm resolution and *TE*/*TR* = 89.5/5, 520ms.

## 3. Results: Dmipy MC-Model Implementations

In this section, we demonstrate the power of Dmipy's modular MC-modeling design. We start by implementing, fitting and showing the results of many well-known MC-models known from the literature. The models we will illustrate are listed in Table 3, where we also indicate each model's composition in terms of biophysical models, the number of lines in Dmipy code it takes to implement, and the primary reference. Note that it is not the point here to deeply study each model's behavior, but to demonstrate how simple it is to create and fit any of them. Deeper, more detailed studies of each model are readily available as examples at the Dmipy Github page at https://github.com/AthenaEPI/dmipy. For each model, we will also denote its schematic, mathematical representation in terms of parameter dependencies, where we will use the “|” sign to separate estimated from fixed parameters during the optimization.

**Table 3:** Overview and composition of implemented MC-models and the number of lines that is needed to implement them from scratch using Dmipy, along with their primary reference.

**Model acronym**	**Model composition**	**No. of lines**	**Primary references**
IVIM (Snip. 8)	2×G1	6	Le Bihan et al., 1988
AxCaliber (Snip. 9)	G1+PD1*C4	8	Assaf et al., 2008
Ball and Sticks (Snip. 10)	G1+N×C1	6	Behrens et al., 2003
NODDI(-x) (Snip. 11)	G1+SD3*(C1+G2)	12	Zhang et al., 2012
Bingham-NODDI (Snip. 11)	G1+SD2*(C1+G2)	12	Tariq et al., 2016
MC-MDI-CSD (Snip. 12)	SD1*(C1+G2)	12	Kaden et al., 2016
MT-CSD (Snip. 13)	2×TR1+TR2	6	Jeurissen et al., 2014
SS3T-CSD (Snip. 14)	2×TR1+TR2	19	Dhollander and Connelly, 2016
ActiveAx (Supplementary Material)	G1+G2+S1+C4	13	Alexander et al., 2010
VERDICT (Supplementary Material)	S4+G1+C1	8	Panagiotaki et al., 2014
SMT (Supplementary Material)	G2	5	Kaden et al., 2015
SMT-NODDI (Supplementary Material)	G1+SD3*(C1+G2)	21	Cabeen et al., 2019
CSD (Supplementary Material)	TR2	6	Tournier et al., 2007

*The MC-models above the midline are presented in the results section of this work, while the implementations of those below the midline are provided in the Supplementary Material*.

### 3.1. IVIM

Intra-voxel incoherent motion (IVIM) is one of the first MC-models used in dMRI (Le Bihan et al., 1988). It uses a 2-compartment model that separates diffusion signal contributions originating from blood flow and Brownian diffusion. The model consists of 2 Ball compartments, each fitting the volume fractions and diffusivities of the blood flow and diffusion, respectively. Changes in e.g., blood volume fraction has been linked to many pathologies such as the vasculature in tumor tissue (Le Bihan, 2017). We represent IVIM in terms of its parameters and the meaning that is assigned to the biophysical models it is composed of, as

(16) $$\begin{array}{r} MC_{\text{IVIM}}=\underset{\text{Blood}}{\underset{︸}{f_{\text{blood}}\overset{\text{Ball}}{\overset{︷}{\text{G1}\left(\cdot |\lambda_{\text{Blood}}\right)}}}}+\underset{\text{Diffusion}}{\underset{︸}{f_{\text{Diffusion}}\overset{\text{Ball}}{\overset{︷}{\text{G1}\left(\lambda_{\text{Diffusion}}\right)}}}}. \end{array}$$

**Figure 4:**
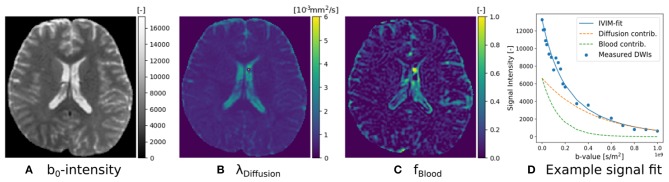
IVIM parameter maps for *S*_0_ **(A)**, λ_Diffusion_ **(B)**, and *f*_blood_ **(C)**, following Equation (16). Notice that the parameter maps are articulate, and the estimated blood volume fraction is significant near the corpus callosum and the sulci. In **(D)** we show an example IVIM fit in a corpus callosum voxel, where we illustrate that the total fitted signal is just the sum of the diffusion and blood signal contributions.

The implementation of Equation (16) is given in Snippet 8. Following recommendations by Gurney-Champion et al. (2016) and Park et al. (2017), we can adjust IVIM's optimization bounds of the diffusion signal diffusivity between [0.5−6] × 10^−9^*m*^2^/*s*, and the blood flow diffusivity between [6−20] × 10^−9^*m*^2^/*s*. Following Gurney-Champion et al. (2018), we can also fix $\lambda_{\text{Blood}}=7\times 10^{-9}m^{2}/s$ to obtain similar results for the remaining parameters. We fit the fixed IVIM model to the IVIM data described in section 2.10.1, and present the parameter maps of an axial brain slice in Figure 4. Notice that signal contributions due to blood flow are significant near the ventricles and the sulci. For a voxel where blood flow and diffusion signal contributions were found to be approximately equal, we also demonstrate the fitted signals of the separate Ball compartments. Notice that blood flow contribution is represented by the fast-decaying, green-dashed line, and the diffusion contribution by the slow-decaying, orange-dashed line.

**Snippet 8:**
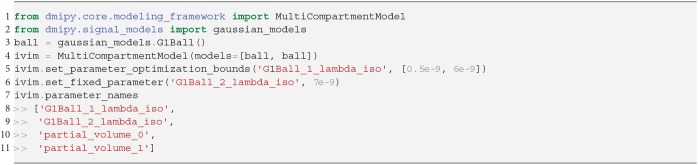
Basic Dmipy implementation of classic IVIM model in 6 lines of code. We set optimization ranges according to suggestions by Park et al. (2017) and Gurney-Champion et al. (2018).

### 3.2. AxCaliber

**Figure 5:**
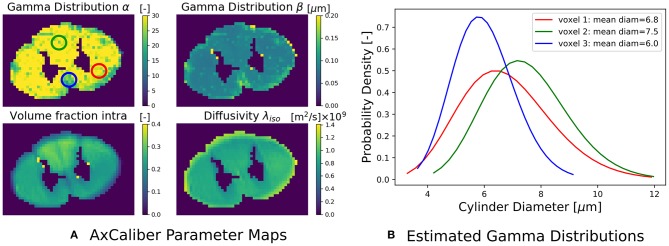
**(A)** The estimated AxCaliber parameter maps. In the map for α, we circled three areas with somewhat different values for α, β, which we use to generate the estimated axon diameter distribution in **(B)**. Notice that while the mean axon diameter of these distributions is similar, the distributions themselves may be quite different.

The estimation of axon diameter from the dMRI signal has been one of the main focuses of dMRI-based microstructure imaging. To this end, we implement the AxCaliber model (Assaf et al., 2008), which models the axon diameter distribution as a set of cylinders with Gamma-distributed diameters. It is given as a two-compartment model as

(17) $$\begin{array}{r} MC_{\text{AxCaliber}}=\underset{\text{Extra-Axonal}}{\underset{︸}{\left(1-f_{\text{intra}}\right)\overset{\text{Ball}}{\overset{︷}{\text{G1}\left(\lambda_{\text{h}}\right)}}}}+\underset{\text{Intra-Axonal}}{\underset{︸}{f_{\text{intra}}\overset{\text{Gamma Distributed Cylinders}}{\overset{︷}{\text{PD1}\left(\alpha ,\beta |\mu ,\lambda_{∥}\right)}}}}, \end{array}$$

where the Ball captures all extra-axonal signal contributions. We note that the cylinder's perpendicular, intra-cylindrical diffusivity is fixed, but it's value is rarely important as for clinically relevant diffusion times the intra-axonal diffusion is restricted anyway (Assaf and Pasternak, 2008). We implement the AxCaliber model in Snippet 9. We use the MIX algorithm to fit AxCaliber to the cat spinal cord data described in section 2.10.2. We chose this data because it has extremely large gradients, which are needed to get enough signal contrast for axon diameter estimation (Drobnjak et al., 2016). As we know the axons are pointing in-plane for the spinal cord, we fixed the orientation of the cylinders to the z-axis, and the parallel diffusivity $\lambda_{∥}=1.7\times 10^{-9}$[*m*^2^/*s*]. We show the resulting parameter maps in Figure 5A. As the results show, the mean axon diameter, estimated as 2αβ of the Gamma distribution, falls within a slightly larger range 3–9 μm than what is known from histology 1–5 μm. This is similar to what Duval et al. (2016) previously found when studying this data with AxCaliber. Even so, even though the mean axon diameter may be similar, we do obtain different diameter distributions as illustrate in Figure 5B.

**Snippet 9:**
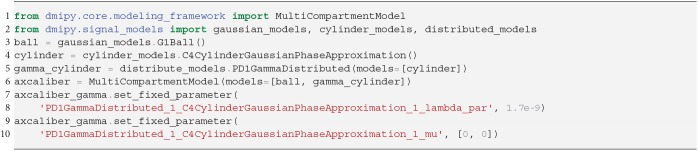
Dmipy implementation of AxCaliber model in 8 lines of code.

### 3.3. Ball and Stick and NODDI Variants

Another main focus of the field has been the estimation of axon orientation dispersion, which is described as the concentration with which the orientation of individual axons is centered around the main bundle axis. In some respects, this tissue property is easier to estimate from the data because it affects the signal at near-clinically feasible gradient strengths (Zhang et al., 2012). The main hypothesis of axon dispersion-based MC-models is that at lower b-values the axon diameter has no effect on the signal, so is assumed to be negligible. The Cylinder model we used in section 3.2 is therefore replaced with a Stick model.

Representing the intra-axonal signal as a Stick, and anything else as Ball, the iconic Ball and Stick (BAS) MC-model was one of the first to estimate the white matter orientation and signal contribution from the dMRI signal (Behrens et al., 2003). Axon dispersion was then modeled by adding a parametric distribution on the orientation of the Stick (Kaden et al., 2007; Sotiropoulos et al., 2013). In particular, using a Watson distribution, in combination with a tortuous Zeppelin to capture the extra-axonal signal, is called the NODDI model (Zhang et al., 2012). Adding multiple dispersed Stick and Zeppelin bundles to represent crossing tissue configurations is called NODDI in crossings (NODDIx) (Farooq et al., 2016), and replacing the Watson with a Bingham distribution is called Bingham-NODDI (Tariq et al., 2016). These MC-model variants are represented as

(18) $$\begin{array}{l} MC_{\text{Sticks}}^{\text{Ball and}}=\underset{\text{Extra-Axonal}}{\underset{︸}{f_{h}\overset{\text{Ball}}{\overset{︷}{\text{G1}\left(\lambda_{\text{iso}}\right)}}}}+\overset{N}{\underset{i=1}{\sum }}\underset{\text{Intra-Axonal}}{\underset{︸}{f_{i,r}\overset{\text{Stick}}{\overset{︷}{\text{C1}\left(\mu_{i}|\lambda_{∥}\right)}}}}, \end{array}$$

(19) $$\begin{array}{r} MC_{\text{Watson}}^{\text{NODDIx}}=\underset{\text{CSF}}{\underset{︸}{f_{\text{CSF}}\overset{\text{Ball}}{\overset{︷}{\text{G1}\left(\cdot |\lambda_{\text{CSF}}\right)}}}}+\overset{N}{\underset{i}{\sum }}\overset{\text{Watson}}{\overset{︷}{\text{SD1}\left(\kappa_{i},\mu_{i}\right)}}{*}_{{𝕊}^{2}}\\ \;[\underset{\text{Hindered Extra-Axonal}}{\underset{︸}{f_{h,i}\overset{\text{Zeppelin}}{\overset{︷}{\text{G2}\left(\cdot |\lambda_{⊥}^{\text{tort}},\lambda_{∥}\right)}}}}+\underset{\text{Intra-Axonal}}{\underset{︸}{f_{r,i}\overset{\text{Stick}}{\overset{︷}{\text{C1}\left(\cdot |\lambda_{∥}\right)}}}}], \end{array}$$

(20) $$\begin{array}{l} MC_{\text{Bingham}}^{\text{NODDI}}=\underset{\text{CSF}}{\underset{︸}{f_{\text{CSF}}\overset{\text{Ball}}{\overset{︷}{\text{G1}\left(\cdot |\lambda_{\text{CSF}}\right)}}}}+\overset{\text{Bingham}}{\overset{︷}{\text{SD2}\left(\kappa_{1},\kappa_{2},\mu ,\psi \right)}}{*}_{{𝕊}^{2}}\\ \;[\underset{\text{Hindered Extra-Axonal}}{\underset{︸}{f_{h}\overset{\text{Zeppelin}}{\overset{︷}{\text{G2}\left(\cdot |\lambda_{⊥}^{\text{tort}},\lambda_{∥}\right)}}}}+\underset{\text{Intra-Axonal}}{\underset{︸}{f_{r}\overset{\text{Stick}}{\overset{︷}{\text{C1}\left(\cdot |\lambda_{∥}\right)}}}}]. \end{array}$$

**Figure 6:**
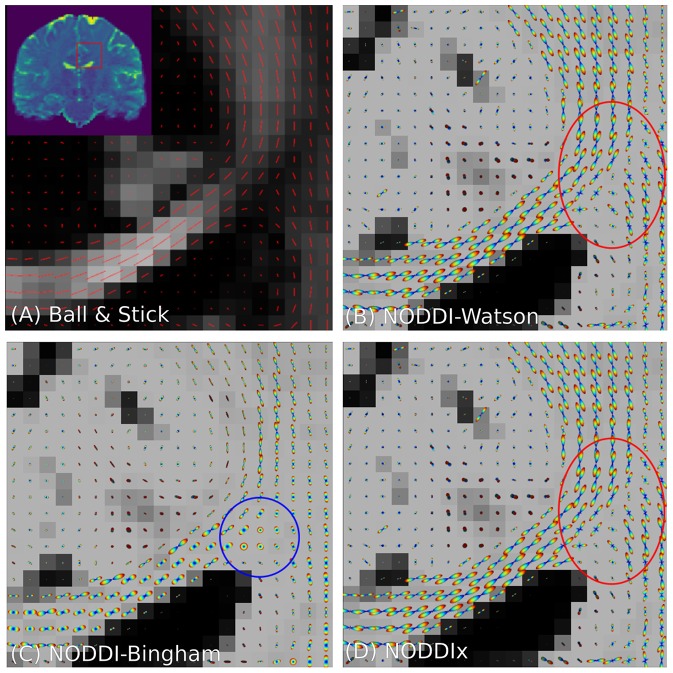
Peak and FOD fields of Ball and Stick **(A)**, NODDI-Watson **(B)**, NODDI-Bingham **(C)**, and NODDIx with two Watson-distributed bundles **(D)** in an area near the centrum semiovale. We expect to find the corpus callosum entering on the left and joining the corona radiata on the right, where we usually find crossing bundle geometries. Models **(A–C)** all appear to correctly reconstruct the orientation of the corpus callosum, but due to their single-bundle design cannot reconstruct crossing structures, instead finding some “average” orientation. It is interesting that NODDI-Bingham, in fact, finds “pancake” FODs to cope with modeling crossing structures (blue circle). NODDIx **(D)**, having two bundles, also accurately reconstructs the corpus callosum despite being over-parameterized for single bundles, and reconstructs some crossings in the centrum semiovale. However, some crossing structures still appear poorly reconstructed (red circle).

It is clear that in terms of MC-model configuration the models in Equations (18–20) are minor variations of each other. We implement BAS in Snippet 10 and the NODDI variants in Snippet 11, and fit them to a subsection of a coronal slice of the HCP data we described in section 2.10.3. We show the main orientation or parametric Fiber Orientation Distributions (FODs) for all models in Figure 6. In all cases, the total signal contribution of the non-Ball compartments is used as background.

**Snippet 10:**
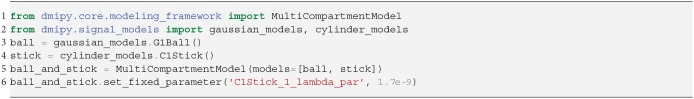
Dmipy implementation of Ball and Stick in 6 lines of code.

**Snippet 11:**
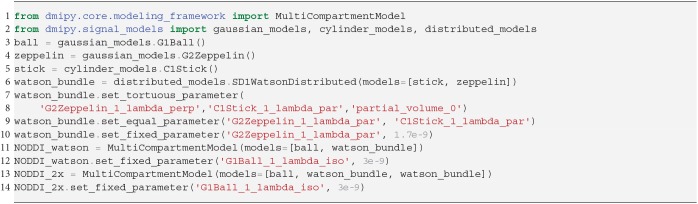
Dmipy implementation of NODDI or NODDIx with 2 bundles in 12 lines of code. The implementation of Bingham-NODDI is equivalent to Watson-NODDI, only replacing SD1WatsonDistributed with SD2BinghamDistributed.

### 3.4. Spherical Mean-Based Constrained Spherical Deconvolution

As we explained in section 2.7.1, spherical mean-based MC-models can estimate dispersion-free tissue parameters by fitting the spherical mean of the measured signal with the spherical mean of the MC-model. To delineate features of the intra- and extra-axonal in the tissue micro-environment, Kaden et al. (2016) proposed the Multi-Compartment Microscopic Diffusion Imaging (MC-MDI) model, composed of the spherical mean of the classic Stick and Zeppelin model with tortuosity constraint. As a cross-modeling framework example, we can then use the voxel-wise estimated MC-MDI parameters to define a voxel-wise varying convolution kernel for an MC-SH model of the same model composition. We describe these two models as

(21) $$\begin{array}{r} \begin{array}{c} \text{Step }1:\;MC_{\text{MC-MDI}}=\underset{\text{Intra-Axonal}}{\underset{︸}{f_{r}\overset{\text{Stick}}{\overset{︷}{{\text{C1}}^{SM}\left(\lambda_{∥}\right)}}}}+\underset{\text{Hindered Extra-Axonal}}{\underset{︸}{\left(1-f_{r}\right)\overset{\text{Zeppelin}}{\overset{︷}{{\text{G2}}^{SM}\left(\lambda_{∥}|\lambda_{⊥}^{\text{tort}}\right)}}}}, \end{array} \end{array}$$

$$\begin{array}{l} \text{Step }2:\;MC_{\text{CSD}}^{\text{vox-varying}}=\text{FOD}\left(\text{c}\right){*}_{{𝕊}^{2}}\\ \;[\underset{\text{Intra-Axonal}}{\underset{︸}{f_{r}^{*}\overset{\text{Stick}}{\overset{︷}{{\text{C1}}^{K}\left(\cdot |\lambda_{∥}^{*}\right)}}}}+\underset{\text{Hindered Extra-Axonal}}{\underset{︸}{\left(1-f_{r}^{*}\right)\overset{\text{Zeppelin}}{\overset{︷}{{\text{G2}}^{K}\left(\cdot |\lambda_{∥}^{*},\lambda_{⊥}^{\text{tort}}\right)}}}}]. \end{array}$$

**Figure 7:**
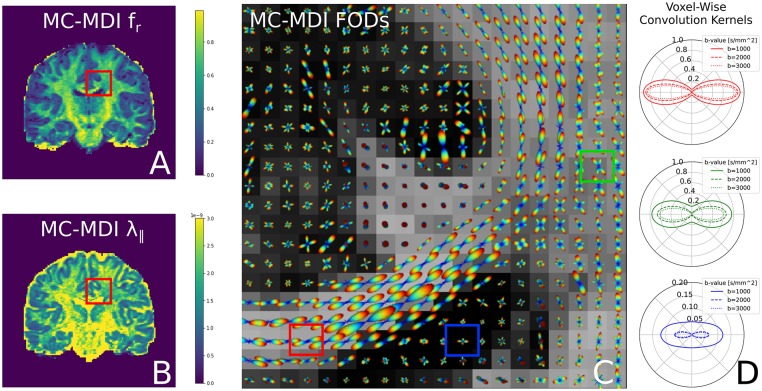
FODs estimated with CSD using varying convolution kernels, estimated voxel-wise using the MC-MDI spherical mean model, see the MC-MDI parameter maps for restricted volume fraction **(A)** and parallel diffusivity **(B)**. In **(C)**, the colored squares highlight three voxels with different tissue compositions; red for a coherent white matter bundle, green for a crossing geometry, and blue for CSF. In **(D)**, we show the corresponding MC-MDI estimated convolution kernels in these voxels for each b-value of the HCP acquisition scheme. Notice that the white matter kernel is more anisotropic than the crossing one, and the CSF kernel is most isotropic and much smaller than the other two. While CSF areas appear more noisy with this voxel-varying kernel approach, it does appear quite effective at resolving crossing structures.

Notice that we use the superscript “*” in step 2 to indicate the fitted parameters of step 1. In the second step, only the spherical harmonics coefficients **c** are estimated, while all the parameters in the convolution kernel are fixed. In fact, using a voxel-varying convolution kernel for CSD-based FOD estimation was the winning approach for the ISMRM 2017 TraCED challenge for reproducible tractography https://my.vanderbilt.edu/ismrmtraced2017/. We fit the 2 steps of our model design to the same coronal slice of the HCP data, and show the FOD field in Figure 7. Notice that on the left we show the *f*_*r*_, λ_∥_ parameters of step 1, in the middle we show the estimated FOD field of step 2, and on the right we show cross-sections of the estimated convolution kernel for three voxels containing different tissue configurations.

### 3.5. Multi- and Single-Shell, Multi-Tissue Constrained Spherical Deconvolution

Dmipy defines MC-SH models as those estimating the non-parametric fiber distribution of some fixed convolution kernel. Unlike the MC-models in the previous sections, the first implementations of MC-SH models were implemented using tissue response models, which are directly estimated from the data, being representative of specific tissue types like white matter (WM), gray matter (GM), and cerebrospinal fluid (CSF) (Tournier et al., 2004, 2007; Jeurissen et al., 2014). In section 3.5.1 we demonstrate how we recover tissue response models directly from the dMRI data. Then, in section 3.5.2, we use these tissue responses to implement and compare Single-Shell 3-tissue CSD with standard Multi-Shell, 3-tissue CSD.

#### 3.5.1. Unsupervised 3-Tissue Response Model Estimation

**Figure 8:**
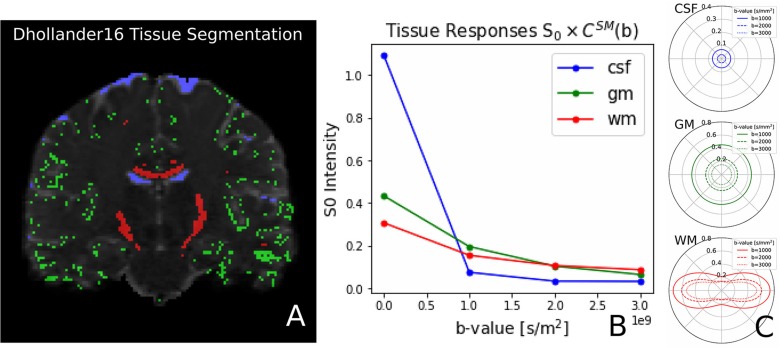
**(A)** Unsupervised three-tissue segmentation algorithm according to Dhollander et al. (2016); **(B)** The spherical mean of the three recovered tissue responses *including* the tissue-specific *S*_0_. notice that the *S*_[0, CSF]_ is three times higher than that of GM and WM. **(C)** The convolution kernels (without *S*_0_) of the three tissues. Notice that WM is the only anisotropic one, and the CSF tissue response is much smaller than the other two.

Recently, Dhollander et al. (2016) proposed an unsupervised method to estimate the GM, WM, and CSF tissue response function directly from the dMRI data. This method is implemented in Dmipy, and we illustrate the results, obtained from the same coronal slice as before, in Figure 8. On the left, we show the gray matter (green), white matter (red), and CSF (blue) segmented voxels, which were found to be the best candidates to represent the three tissue types. In the middle, we show the spherical mean of the three recovered tissue responses *including* the tissue-specific *S*_0_ intensities. On the right, we show the recovered kernel cross-sections without the *S*_0_ intensities. Notice that the CSF *S*_0_ intensity makes the CSF signal contribution much larger than the others at *b*_0_, but it quickly becomes negligible at higher *b*-values.

#### 3.5.2. Comparing Single and Multi-Shell, Multi-Tissue CSD

Having recovered the tissue response functions, we can now define the standard Multi-Tissue Constrained Spherical Deconvolution (MT-CSD) model (Jeurissen et al., 2014).

(22) $$\begin{array}{l} S_{\text{MT-CSD}}=\underset{\text{CSF}}{\underset{︸}{S_{0,\text{CSF}}f_{\text{CSF}}\overset{\text{Iso Response}}{\overset{︷}{\text{TR1}\left(\cdot \right)}}}}+\underset{\text{Gray Matter}}{\underset{︸}{S_{0,\text{GM}}f_{\text{GM}}\overset{\text{Iso Response}}{\overset{︷}{\text{TR1}\left(\cdot \right)}}}}\\ \;+\underset{\text{White Matter}}{\underset{︸}{S_{0,\text{WM}}f_{\text{WM}}\overset{\text{Fiber Dist.}}{\overset{︷}{\mathrm{FOD}\left(\text{c}\right)}}{*}_{{𝕊}^{2}}\overset{\text{Aniso Response}}{\overset{︷}{{\text{TR2}}_{\text{WM}}\left(\cdot \right)}}}} \end{array}$$

Notice that in contrast to the previous models, MT-CSD is *signal-based*, including the tissue-specific *S*_0_ intensities. By not normalizing the *b*_0_ intensity of each model to unity, we can include the signal information in the *b*_0_ image as another “shell” to estimate information from. This is exactly what Dhollander and Connelly (2016) takes advantage of when they proposed so-called Single-Shell 3-Tissue (SS3T) CSD.

In SS3T, it is possible to obtain multi-shell-like parameter maps from “single”-shell data, that resemble those of multi-shell MT-CSD. This is counter-intuitive, as it seems impossible to fit three compartments from only two shells. As we will show, the trick of the SS3T algorithm lies in that it uses an iterative, partial optimization scheme based on parameter cascading, starting from a very specific initialization of the MT-CSD. First, they fix *f*_WM_ = 0 and optimize only *f*_GM_ and *f*_CSF_. Then, they fix *f*_CSF_ to the previous solution, and only optimize *f*_WM_ and *f*_GM_. The process is repeated by then fixing *f*_WM_ to the previous solution and so on, for a *fixed* amount of iterations, where Dhollander and Connelly (2016) used 4. Dmipy implements this meta-algorithm in Snippet 14.

**Snippet 12:**
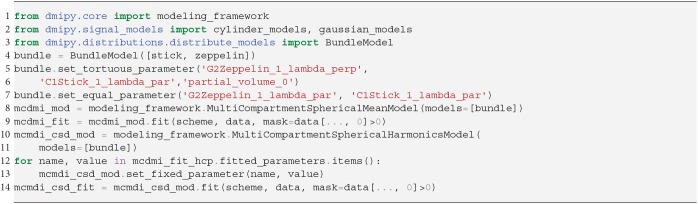
Dmipy implementation of 2-step MC-MDI to voxel-varying CSD in 12 lines of code.

**Snippet 13:**
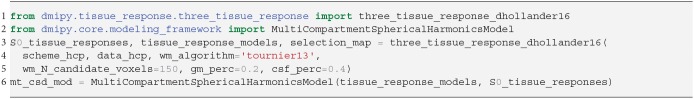
Dmipy implementation of unsupervised tissue response estimation and Multi-Tissue CSD in 6 lines of code.

**Snippet 14:**
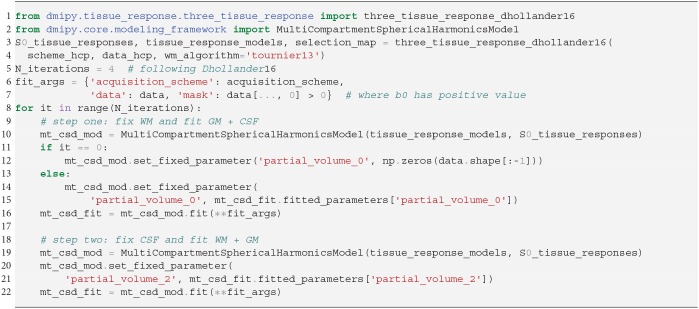
Dmipy implementation of Single-Shell Three-Tissue (SS3T) Meta-Algorithm in 19 lines (ignoring commenting lines).

**Figure 9:**
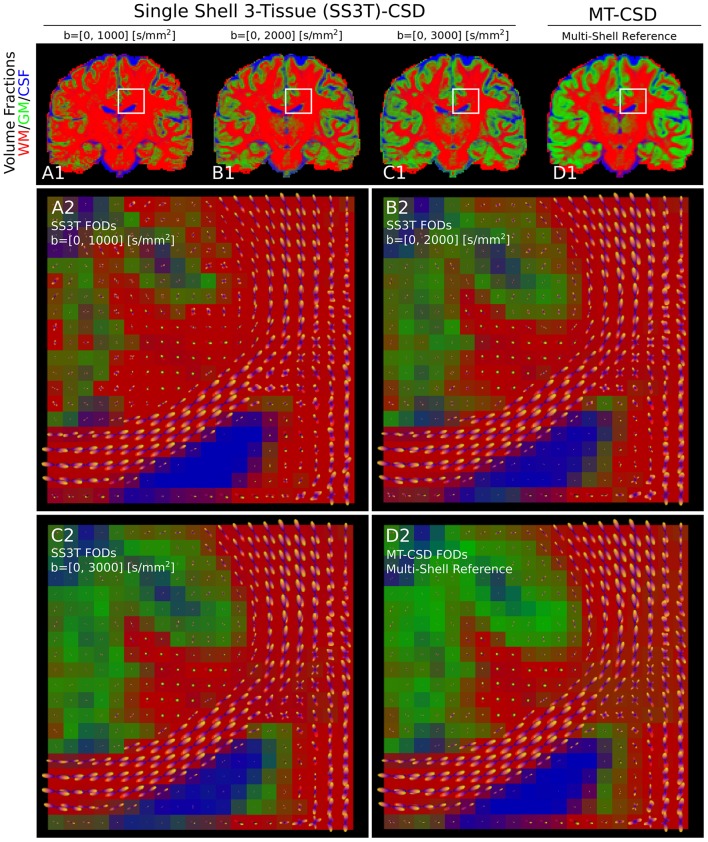
**(A1–D1)** Single-shell three-tissue (SS3T) volume fraction comparison with the standard multi-shell CSD (MT-CSD) implementation. From left to right, the SS3T algorithm is applied to higher b-shell data. Notice that as higher b-value data is used, the gray matter fraction becomes more prominent, coming close to resembling the MT-CSD results for the b=[0,3,000] [s/mm^2^] data. In **(A2–D2)** we illustrate the corresponding FODs for each SS3T and MT-CSD optimization. Notice that the crossing appears better resolved at higher b-values (as expected), but the gray matter fraction is significantly suppressed at lower b-values.

Applying SS3T then to the whole coronal slice of the HCP data, we can visualize the volume fractions as RGB values in Figure 9. Notice that as the single-shell data has a higher *b*-value, the SS3T results more closely approximate the multi-shell MT-CSD reference. On the bottom, we visualize the estimated FODs with the RGB volume fraction values as background. Notice that higher *b*-value SS3T results seem to resolve the crossing slightly better, but that the biggest difference can be seen in the lower GM volume fractions for lower *b*-values.

## 4. Discussion

This work introduces Dmipy, an open-source software solution for generalized dMRI signal modeling and Microstructure Imaging using custom-designed multi-compartment models. The toolbox is freely available at https://github.com/AthenaEPI/dmipy. In this section, we discuss Dmipy's contributions to multi-compartment-based microstructure research.

### 4.1. Generalized, On-the-Fly Multi-Compartment Modeling and Signal Generation

Dmipy's key contribution is the way it generalizes dMRI signal modeling regardless of the MC-model composition or acquisition scheme design. In this way, Dmipy can be better seen as a “model generator,” i.e., it does not hard-code any model combinations, but can generate any one of them on the fly. By standardizing the interaction between the PGSE acquisition scheme representation and the mathematical representations of any biophysical model, all signal representations become the same from a user point of view. An MC-model can then be seen as nothing more than an aggregated set of the input model parameters, including volume fractions, which is neatly represented in Equation (3). In this way, Dmipy enables the user to have only high-level interaction in choosing which model is appropriate the chosen application and never has to worry about the underlying mathematical implementation.

Throughout this work we have provided the basic commands in Snippets that allows easy interaction with all aspects of MC-modeling. In particular, we can load the acquisition parameters for any PGSE sequence (Snippet 1), generate the dMRI signal for any individual compartment model (Snippet 2), design any MC-model variant and set *S*_0_ responses (Snippet 5), define parameter links (Snippet 6), and fit an MC-model to data (Snippet 7), from which the fitted model parameters and fitting error can be obtained. Using these elementary commands, we can implement and fit many well-known MC-models from literature in around 10 lines of code (see Table 3). In section 3, we explicitly provided the Dmipy code and described the estimated model parameters of IVIM (Le Bihan et al., 1988), AxCaliber (Assaf et al., 2008), Ball and Stick (Behrens et al., 2003), various forms of NODDI (Zhang et al., 2012; Farooq et al., 2016; Tariq et al., 2016), spherical-mean-based MT-CSD (Jeurissen et al., 2014) and spherical-harmonics-based MC-MDI (Kaden et al., 2016). For completeness, in the Supplementary Material, we also provide the implementations of ActiveAx (Alexander et al., 2010), VERDICT (Panagiotaki et al., 2014), SMT (Kaden et al., 2015), SMT-NODDI (Cabeen et al., 2019), and CSD (Tournier et al., 2007). Extended explorations of these models can also be found at the Dmipy website.

### 4.2. Multi-Compartment Spherical Mean (MC-SM) Modeling

Dmipy's generalization of MC-modeling also extends to alternative modeling frameworks, including spherical mean modeling (MC-SM) in section 2.7.1. With respect to MC-SM, its key interest is that by taking the shell-wise spherical mean of the signal and the compartment model, the effects of axon dispersion or crossing bundle geometries on the signal is obviated. Kaden et al. (2016) showed using such MC-SM models one could recover a voxel-varying, parametric representation of the undispersed tissue parameters. Dmipy generalizes the spherical mean representation to all three-dimensional compartment models—including those without closed-form like Cylinders and Spheres (section 2.5.2). In previous work, we explored this in the setting of spherical mean-based axon diameter estimation (Pizzolato et al., 2018). See the Supplementary Material for the implementation details.

### 4.3. Multi-Compartment Spherical Harmonics (MC-SH) Modeling

In section 2.7.2, we showed that Dmipy's MC-modeling generalization also extends to models based on spherical convolution by means of spherical harmonics (SH). The key interest in such MC-SH models is that the Fiber Orientation Distribution (FOD) of any fixed convolution kernel can be estimated in a model-free way (Tournier et al., 2004). Dmipy generalizes the interface by which the type and number of convolution kernels can be defined in MC-SH models, allowing for mixes of both parametric and non-parametric compartment models. In this way, it can be seen as a generalized implementation the Multi-Tissue Constrained Spherical Deconvolution (MT-CSD) framework by Jeurissen et al. (2014).

### 4.4. Generalized Implementation of Tissue Response Models

Tissue Response models are have been used in literature to get an approximation of the shape and amplitude of the signal in specific tissue segmentations—typically white matter, gray matter, and CSD (Tournier et al., 2007; Jeurissen et al., 2014). Different algorithms have been proposed to recover the most appropriate white matter response, either by looking at DTI's FA map (Tournier et al., 2007) or by iterative means (Tournier et al., 2013; Tax et al., 2014). Dmipy implements both these approaches, including the recovery of all three tissue types directly from dMRI data using the unsupervised algorithm by Dhollander et al. (2016) (see Figure 8). But, more importantly, Dmipy provides a generalized theory to create anisotropic or isotropic tissue responses, just by providing the data from segmented voxels, in section 2.6.3. In this way, both the tissue response and the tissue-specific *S*_0_ intensity can be recovered for any user-defined segmentation, opening the paths to multi-tissue modeling beyond the three classic WM, GM, and CSF tissues. Moreover, tissue responses can be given to all MC-modeling variants, opening the door to Multi-Tissue modeling for MC-models other than MT-CSD (Jeurissen et al., 2014).

### 4.5. Modular, Generalized Optimization Algorithms

By unifying the theory of MC, MC-SM, and MC-SH modeling, by extension Dmipy allows generalized implementations of standard or dMRI-specific data fitting algorithms, based on Equations (3, 10, 12). Calling different optimization algorithms can be done using a single line of code (see Snippet 7). For MC and MC-SM models, Dmipy uses Scipy (Jones et al., 2001) to implement the “brute2fine” optimizer, using standard brute-force to find a good starting point, and then using L-BFGS-B (Byrd et al., 1995) to refine the solution to a local minimum. Enforcing the unity constraint on the volume fraction is done by nesting the volume fractions based on works by Zhang et al. (2012). We also implement the recent MIX algorithm by Farooq et al. (2016), taking advantage of Scipy's differential evolution algorithm (Storn and Price, 1997). For MC-SH models, Dmipy uses Numpy (Oliphant, 2006) and CVXPY (Diamond and Boyd, 2016; Agrawal et al., 2018) to implement classic Tikhonov-regularized CSD (Tournier et al., 2007) and Multi-Tissue CSD (Jeurissen et al., 2014). However, it should be noted that Dmipy's optimizers are modular, and thus alternative optimization algorithms can be easily plugged in. We provide details on the implementation of the optimization algorithms in the Supplementary Material.

### 4.6. Enabling Iterative, Cross-Framework Modeling Through Uniform Parameter Definitions

By generalizing the meaning of parameters over MC-modeling frameworks, it follows we can initialize the parameters to be optimized in one MC-model with the parameters obtained from another MC-model. Harms et al. (2017) used this concept to initialize more complicated MC-models with the obtained values of simpler MC-models. In Dmipy, we take this a step further, in that we can take advantage of the unique properties of different MC-modeling frameworks. In section 3.4, we use this concept to first estimate the parameters of spherical mean-based MC-DMI model (Kaden et al., 2016). Then, we use optimized parameters of MC-MDI to define a spherical harmonics-based Multi-Compartment CSD model that uses a voxel-varying convolution kernel to estimate the FOD for each voxel. We illustrate the results in Figure 7, where we can notice how well crossings are resolved. In fact, this was also the winning approach for the ISMRM 2017 TraCED challenge for reproducible tractography https://my.vanderbilt.edu/ismrmtraced2017/, which can now be easily implemented and further explored using Dmipy.

This process can also be exploited within the same MC-modeling framework, even within the same MC-model. In section 3.5, we implement the state-of-the-art Single-Shell Three-Tissue (SS3T) algorithm in Snippet 14. As we illustrate in Figure 9, the SS3T algorithm is able to obtain “multi-shell-like” MT-CSD results by only using one DWI shell (plus a *b*_0_). It is based on premature termination of an iterative optimization process, whose parameters *briefly* look like the multi-shell solution by virtue of the chosen initialization point. Though, it appears this algorithm works better on higher b-shell data.

### 4.7. Limitations of the Dmipy Toolbox

At this moment, Dmipy only implements biophysical models designed for the PGSE-dMRI pulse sequence. The main reason for initially targeting the PGSE sequence is because it is currently still the most used sequence, and high-quality PGSE-dMRI data is widely available from excellent sources like the Human Connectome Project. However, Dmipy's modular philosophy may also be applied to MC-models based other sequences, like double-diffusion encoding (Coelho et al., 2019), free waveforms (Ianuş et al., 2016) or multi-dimensional MRI (Nilsson et al., 2018), if only the appropriate acquisition scheme and biophysical model formulations were implemented. All subsequent steps, i.e., combining, fitting, and analyzing the fitted parameters of an MC-model, are already dMRI-sequence agnostic in Dmipy.

### 4.8. Improving Research Reproducibility Through High Coding Standards

Dmipy provides a concrete means by which dMRI-based microstructure researchers can easily construct and fit the models that are appropriate for their application. Furthermore, Dmipy's user-friendly, building-block-based coding style makes it easy to reliably construct and apply the same MC-model and optimization algorithm, removing the need for researchers to spend time implementing and testing the underlying algorithms. To ensure high-quality coding standards, we currently have over 83% testing coverage and follow Google's style guide for consistent readability and maintenance. In this way, Dmipy contributes to open-source, highly reproducible microstructure research.

## 5. Conclusion

The open-source Dmipy framework represents the unification and implementation of dMRI-based MC-modeling over the last decades. By adopting a “building-block” based philosophy in both the theory and the coding framework, Dmipy is highly modular and is able to construct on-the-fly MC-models in around 10 lines of code. Furthermore, “standard” MC-modeling is generalized with spherical mean-based and spherical convolution-based MC-modeling, allowing for new, cross-MC-modeling approaches. Importantly, Dmipy's key innovation is not that it proposes new methods, but that it generalizes and combines the advantages of already existing works^4^. By providing a well-tested, user-friendly toolbox that simplifies the interaction with the otherwise complicated field of dMRI-based Microstructure Imaging, Dmipy contributes to more reproducible, high-quality research.

## Data Availability Statement

The code and all results in this work can be completely reproduced using the tools available at the Dmipy repository at https://github.com/AthenaEPI/dmipy. The Human Connectome Project (HCP) data used to illustrate several of the algorithms is available at https://db.humanconnectome.org/. The spinal cord data is available at the White Matter Microscopy Database at https://dipy.org. The IVIM dataset was downloaded through the Dipy example at http://nipy.org/dipy/examples_built/reconst_ivim.html.

## Ethics Statement

Ethical review and approval was not required for the study on human participants in accordance with the local legislation and institutional requirements. The patients/participants provided their written informed consent to participate in this study.

## Author Contributions

RF devised the project, developed the Dmipy software to its mature form, and wrote the manuscript. DW designed the seminal code for the Dmipy framework and restricted signal models. RD supported this project and co-proposed and co-supervised it with DW. RF, DW, and RD discussed the framework and results, provided critical feedback, helped shape the research, analysis, and final manuscript.

### Conflict of Interest

RF was employed by company TheraPanacea. The remaining authors declare that the research was conducted in the absence of any commercial or financial relationships that could be construed as a potential conflict of interest.

## Footnotes

^1^http://mig.cs.ucl.ac.uk

^2^http://www.mrtrix.org/

^3^https://nipy.org/dipy/

^4^Embodying Newton's famous saying “If I have seen further it is by standing on the shoulders of Giants.”
