# Supplementary material for: The Dmipy Toolbox: Diffusion MRI Multi-Compartment Modeling and Microstructure Recovery Made Easy
Source: Front Neuroinform. 2019 Oct 15;13:64. doi: 10.3389/fninf.2019.00064 (PMC6803556; doi:10.3389/fninf.2019.00064)
Supplement: Supplementary file 1 [file Data_Sheet_1.PDF]

frontiers

Supplementary Material

1 SPHERICAL HARMONICS AND SPHERICAL CONVOLUTION

1.1 Signal Approximation with Spherical Harmonics

Spherical Harmonics (SH), normally indicated by Y_l^m (l denotes the order and m the phase factor), is a basic for complex functions on the unit sphere. Explicitly, they are given as follows

$$Y_l^m(\theta, \phi) = \sqrt{(\frac{2l+1}{4\pi}\frac{(l-m)!}{(l+m)!})}P_l^m(\cos\theta)e^{im\phi} \tag{S1}$$

where (θ, ϕ) obey physics convention ($\theta \in [0, \pi], \phi \in [0, 2\pi]$) and P_l^m is an associated Legendre polynomial. Following Descoteaux et al. (2007), for $l = 0, 2, 4, \ldots, l_{\text{max}}$ and $m = -l, \ldots, 0, \ldots, l$, we define the new index $j := (j(l, m) = (l^2 + l + 2)/2 + m$ and define our modified basis $\mathbf{Y}$ with elements Y_j such that

$$Y_j = \begin{bmatrix} \sqrt{2}\cdot\text{Re}(Y_l^k) & \text{if} -l \leq m < 0 \\ Y_l^0 & \text{if} m = 0 \\ \sqrt{2}\cdot\text{Img}(Y_l^k) & \text{if} 0 < m \leq l \end{bmatrix} \tag{S2}$$

where $\text{Re}(Y_l^m)$ and $\text{Img}(Y_l^m)$ represent the real and imagnary parts of Y_l^m, respectively. The basis is designed to be symmetric, real and orthonormal. We thus approximate the signal, for a certain shell, at each of the N gradients i as

$$S(\mathbf{n}_i) = S(\theta_i, \phi_i) = \sum_{j=1}^{N_\mathbf{c}} c_j Y_j(\theta_i, \phi_i), \tag{S3}$$

where $N_\mathbf{c} = (l_{\text{max}} + 1)(l_{\text{max}} + 2)/2$ is the number of terms in the modified SH basis $\mathbf{Y}$ of order l. Letting $\mathbf{y}$ be the $N_s \times 1$ vector representing the input signal of every encoding gradient direction of shell s, $\mathbf{c}$ the $N_\mathbf{c} \times 1$ vector of SH coefficients c_j and Ψ is the $N_s \times N_\mathbf{c}$ matrix constructed with the discrete modified SH basis

$$\Psi = \begin{bmatrix} Y_1(\mathbf{n}_1) & Y_2(\mathbf{n}_1) & \ldots & Y_{N_\mathbf{c}}(\mathbf{n}_1) \\ \vdots & \vdots & \ddots & \vdots \\ Y_1(\mathbf{n}_{N_s}) & Y_2(\mathbf{n}_{N_s}) & \ldots & Y_{N_\mathbf{c}}(\mathbf{n}_{N_s}) \end{bmatrix} \tag{S4}$$

we can write the set of equations as an over-determined linear system $\mathbf{y} = \Psi\mathbf{c}$. To estimate the SH coefficients from the measures signal, we can thus invert this equation such that $\mathbf{c} = \Psi^\dagger\mathbf{y}$, where † represents the Penrose pseudo-inverse.

1.2 Spherical Mean from Spherical Harmonics

While it is hard to properly calculate the spherical mean of the signal from the discrete measurements, it is simple to do so using the spherical harmonics expansion. In fact, after $\mathbf{c}$ has been recovered, then the spherical mean is given by $2\sqrt{\pi}\,\mathbf{c}_{m=0,l=0}$, where the $2\sqrt{\pi}$ is the jacobian on the sphere.

1.3 Single-Shell, Single-Compartment Spherical Convolution

As spherical harmonics are analogous to the spherical Fourier transform, they provide the same benefits advantages when it comes to spherical convolution: A convolution of two spherical functions in the regular space can instead be performed by multiplying their SH coefficients. For this reason, one of the main applications of spherical harmonics in dMRI is to facilitate the spherical convolution of some spherical distribution function $\mathrm{F}(\mathbf{n}) : \mathbb{S}^2 \rightarrow [0, \infty]$ with some spherical kernel function $K(\mathbf{n}) : \mathbb{S}^2 \rightarrow [0, \infty]$, given continuously by

$$S(\mathbf{n}) = \int_{\mathbb{S}^2} \mathrm{F}(\mathbf{n} - \mathbf{u}) \times K(\mathbf{u}) d\mathbf{u} \tag{S5}$$

To perform the spherical convolution using spherical harmonics, the F must be transformed to spherical harmonics, and K must be transformed to *rotational* harmonics. Rotational harmonics are a special case of spherical harmonics where the spherical profile is axially symmetric and aligned along the Z-axis. In this case, the profile can be described using only the even $l, m = 0$ SH coefficients. For a given spherical and rotational harmonics expansion of F and K, the convolution Eq. (S5) can be given by

$$\hat{S}_{l[j],m[j]} = \sqrt{\frac{4\pi}{(2l[j]+1)}} \hat{\mathrm{F}}_{l[j],m[j]} \hat{K}_{l[j],m=0} \tag{S6}$$

where the first term represents the Eigenvalues of the SH expansion, see e.g. `https://www.cs.jhu.edu/~misha/Spring15/17.pdf`, slide 59-60.

Assuming the convolution kernel is known, we are interested in recovering the convolved signal S in the normal space using a single matrix-vector multiplication with the SH coefficients of distribution F. This can be achieved by combining Eqs. (S4) and (S6) to create spherical convolution matrix $\mathbf{M}$ of shape $N_s \times N_{\mathbf{c}}$ such that

$$\mathbf{y}^{SS} = \mathbf{M}\mathbf{c} \quad \text{with} \quad \mathbf{M}[i,j] = \sqrt{\frac{4\pi}{(2l[j]+1)}} \hat{K}_{l[j],m=0} Y_{l[j],m[j]}(\mathbf{n}[i]). \tag{S7}$$

1.4 Multi-Shell, Single-Compartment Spherical Convolution

So far, we've been considering that the measured signal, kernel and FOD all live on a single spherical shell. In dMRI, however, the signal and kernel can be measured at multiple shells s at the same time. As the spherical convolution in Eq. (S7) must be applied *separately* to each shell, but for the same FOD coefficients, we can create a multi-shell convolution matrix as a horizontal concatenation of single-shell convolution matrices. Let $\mathbf{y}^{MS} = [\mathbf{y}_1, \ldots, \mathbf{y}_{N_s}]$ be the multi-shell signal as a list of the measurements on different shells s. The multi-shell convolution is then given by

$$\mathbf{y}^{MS} = \mathbf{M}^{MS}\mathbf{c} \quad \text{with} \quad \mathbf{M}^{MS} = [\mathbf{M}_1, \ldots, \mathbf{M}_{N_s}], \tag{S8}$$

where each single-shell convolution matrix $\mathbf{M}_s$ has been computed using the orientations $\mathbf{n}$ and kernel coefficients $\hat{K}$ of shell s.

1.5 Multi-Shell, Multi-Compartment Spherical Convolution

As a last generalization, we consider the case where are representing the multi-shell measured signal S as a linear sum of N_{mod} spherical convolutions. In this case, we can expand the multi-shell convolution in Eq. (S8) to

$$\mathbf{y}^{\text{MC-MS}} = \mathbf{M}^{\text{MC-MS}}\mathbf{c}^{\text{MC-MS}} = \begin{bmatrix} \mathbf{M}_1^{MS} \\ \vdots \\ \mathbf{M}_{N_{mod}}^{MS} \end{bmatrix} \begin{bmatrix} \mathbf{c}_1 \\ \vdots \\ \mathbf{c}_{N_{mod}} \end{bmatrix}, \tag{S9}$$

where each $\mathbf{M}_n^{MS}$ is generated according to the multi-shell formulation, with the rotational harmonics coefficients of kernel n. Notice that the multi-compartment spherical harmonics coefficients $\mathbf{c}^{\text{MC-MS}}$ are just a concatenation of the coefficients of the single-model coefficients $\mathbf{c}_n$.

1.6 Single- and Multi-Compartment, Multi-Shell Spherical Deconvolution

Spherical deconvolution is performed to find the best distribution F that distributes the kernel K, such that the resulting modeled signal best fits the measured signal. The only thing that needs to be known is the representation of the kernel K, and the expansion order of the spherical harmonics. The obtained FOD distribution, represented in $\mathbf{c}$, must also be positive. Formally, the FOD estimation for a single kernel (compartment) is given by

$$\mathbf{c}^* = \text{argmin}_{\mathbf{c}} \|\mathbf{y} - \mathbf{M}^{\text{MS}}\mathbf{c}\|^2 \quad \text{s.t.} \quad \Phi\mathbf{c} \geq 0 \tag{S10}$$

where $\mathbf{M}^{\text{MS}}$ is given in Eq. (S8) and Φ maps the SH coefficients $\mathbf{c}$ to a dense mapping on the sphere. The generalization to multi-compartment spherical deconvolution is given by

$$\mathbf{c}^* = \text{argmin}_{\mathbf{c}} \|\mathbf{y} - \mathbf{M}^{\text{MC-MS}}\mathbf{c}^{\text{MC-MS}}\|^2 \quad \text{s.t.} \quad \Phi_n\mathbf{c}_n \geq 0 \tag{S11}$$

where $\mathbf{M}^{\text{MC-MS}}$ is given in Eq. (S9), and Φ_n maps the FOD coefficients $\mathbf{c}_n$ for kernel n to a dense spherical sampling.

2 MATHEMATICAL REPRESENTATION OF PARAMETRIC BIOPHYSICAL MODELS AND DISTRIBUTIONS

In Dmipy, models are either isotropic, or anisotropic but axially symmetric. Following Assaf et al. (2004), anisotropic models can be written as a separable product of parallel and perpendicular components, such that

$$C_{\text{iso}}(\mathbf{A}, \mathbf{p}) = C(\mathbf{A}, \mathbf{p}) \tag{S12}$$

$$C_{\text{aniso}}(\mathbf{A}, \mathbf{p}_{||}, \mathbf{p}_{\perp}) = C_{||}(\mathbf{A}_{||}, \mathbf{p}_{||}) \times C_{\perp}(\mathbf{A}_{\perp}, \mathbf{p}_{\perp}), \tag{S13}$$

where the parallel and perpendicular acquisition schemes $\mathbf{A}_{||}$ and $\mathbf{A}_{\perp}$ differ from the measured $\mathbf{A}$ in that the gradient vector $\mathbf{G} = G\mathbf{n}$ is projected parallel and perpendicular to $\boldsymbol{\mu}$, such that $G_{||} = \mathbf{G}^T\boldsymbol{\mu}$, and $G_{\perp} = (\mathbf{G}^T\mathbf{G} - (\mathbf{G}^T\boldsymbol{\mu})^2))^{1/2}$. This operation is analogous for b and q.

Table S1. Restricted Model Assumptions and principal references. Dmipy implements Planes (P), Cylinders (C), and Spheres (S). We number the approximations consistently across geometries: 2 for short gradient pulse approximation and long diffusion time regime; 3 for long diffusion diffusion time regime only; and 4 for Gaussian Phase Approximation (GPA).

Acquisition Assumptions	Dmipy Model Reference	Principal Reference
$\delta \to 0, \Delta \gg R^2/D$	P2	(Balinov et al., 1993)
	C2	(Söderman and Jönsson, 1995)
	S2	(Balinov et al., 1993)
$\delta \to 0$	P3	(Callaghan, 1995)
	C3	(Callaghan, 1995)
	S3	(Callaghan, 1995)
GPA	P4	(Balinov et al., 1993)
	C4	(Vangelderen et al., 1994)
	S4	(Murday and Cotts, 1968)

Dmipy's toolbox contains parametric representations of many biophysical models and distributions, can can be used in MC-model design. Here, we provide the explicit equations of restricted models in 2.1, spherical distributions in 2.2 and spatial distributions in 2.3.

2.1 Restricted Models

In Table S1 we provide the acquisition assumptions, Dmipy model references and principal reference in literature for the restricted models for Planes, Cylinders and Planes. In Table S2 we provide equations for each representation. Please refer to the principal references for the meanings of all symbols, but notice that the more assumptions there are on the PGSE acquisition, the simpler the mathematical representation.

2.2 Parametric Spherical Distributions

To provide a more straightforward, but less general way to quantify axon dispersion, parametric representations for F such as the Bingham and Watson distribution have been proposed (Kaden et al., 2007).

The Bingham distribution $\mathrm{B}(\mathbf{n}|\boldsymbol{\mu},\kappa_1,\kappa_2)$ is an antipodally symmetric distribution, centered around direction $\boldsymbol{\mu}$, describing a possibly anisotropic density with concentration parameters κ_1 and κ_2 (Bingham, 1974). More formally, its probability density along normalized unit vector $\mathbf{n}$ is given as

$$\mathrm{B}(\mathbf{n}|\boldsymbol{\mu},\kappa_1,\kappa_2,\psi)=\frac{\exp(\mathbf{n}^T\mathbf{B}\mathbf{n})}{4\pi\,{}_1F_1(1/2;3/2;\mathbf{B})}\quad\text{with}\quad\mathbf{B}=\mathbf{R}^T\mathbf{B}_{\text{diag}}\mathbf{R}\tag{S14}$$

with ${}_1F_1$ the confluent hypergeometric function, $\mathbf{R}$ a rotation matrix that aligns the distribution with $\boldsymbol{\mu}$ and $\mathbf{B}_{\text{diag}}=\text{Diag}(\kappa_1,\kappa_2,0)$. Note that concentration parameters κ_1,κ_2 are inversely related to dispersion. The parameters of B can only be obtained by non-linear estimation from Eq. (S5), but by giving up the ease of estimation using SH and ability to describe multiple bundles, we now directly estimate parameters that are related to axon dispersion. The Watson distribution $W(\mathbf{n}|\boldsymbol{\mu},\kappa)$ is a special case of Bingham when $\kappa=\kappa_1=\kappa_2$, meaning $W(\mathbf{n}|\boldsymbol{\mu},\kappa)=B(\mathbf{n}|\boldsymbol{\mu},\kappa,\kappa)$.

$$\mathrm{W}(\mathbf{n}|\boldsymbol{\mu},\kappa)=\frac{\exp\left(\kappa(\boldsymbol{\mu}\cdot\mathbf{n})^2\right)}{4\pi\,{}_1F_1(1/2;3/2;\kappa)}\tag{S15}$$

Table S2. Signal approximations for diffusion inside planes (P), cylinders (C) and spheres (S) for different PGSE acquisition scheme assumptions. In all cases the signal represents the diffusion perpendicular to the geometry boundary.

Acq. Ass.		Representation
$\delta\rightarrow 0, \Delta \gg R^2/D$	P2:	$\frac{2\left(1-\cos(4\pi qR)\right)}{(4\pi qR)^2}$
	C2:	$\left(\frac{J_1(2\pi qR)}{\pi qR}\right)^2$
	S2:	$\left(\frac{3}{(2\pi qR)^2}\left(\frac{\sin(2\pi qR)}{(2\pi qR)}-\cos(2\pi qR)\right)\right)^2$
$\delta\rightarrow 0$	P3:	$\sum_{n=0}^{\infty}\exp\left[-\frac{\xi_m^2 D\Delta}{a^2}\right]\frac{2[(2\pi qa)\sin(2\pi qa)\cos(\xi_n)-\xi_n\cos(2\pi qa)\sin(\xi_n)]^2}{(1+\sin(2\xi_n)/2\xi)[(2\pi qa)^2-\xi_n^2)]^2} + \sum_{m=0}^{\infty}\exp\left[-\frac{\zeta_m^2 D\Delta}{a^2}\right]\frac{2[(2\pi qa)\cos(2\pi qa)\sin(\zeta_m)-\zeta_m\sin(2\pi qa)\cos(\zeta_m)]^2}{(1-\sin(2\zeta_m)/2\zeta_m)[(2\pi qa)^2-\zeta_m^2]^2}$
	C3:	$\sum_{k}^{\infty}4\exp(-\beta_{0k}^2 D\Delta/R^2)\times\frac{\left((2\pi qR)J_0'(2\pi qR)\right)^2}{\left((2\pi qR)^2-\beta_{0k}^2\right)^2} + \sum_{nk}^{\infty}8\exp(-\beta_{nk}^2 D\Delta/R^2)\times\frac{\beta_{nk}^2}{\left(\beta_{nk}^2-n^2\right)}\times\frac{\left((2\pi qR)J_n'(2\pi qR)\right)^2}{\left((2\pi qR)^2-\beta_{nk}^2\right)^2}$
	S3:	$\sum_{nk}6\exp\left[-\frac{\alpha_{nk}^2 D\Delta}{a^2}\right]\frac{(2n+1)\alpha_{nk}^2}{\frac{1}{4}+\alpha_{nk}^2-(n+\frac{1}{2})^2}\times\frac{[(2\pi qa)j_n'(2\pi qa)]^2}{[(2\pi qa)^2-\alpha_{nk}^2]^2}$
GPA	P4	$\exp\left(-\frac{8\gamma g^2 a^4}{D\pi^6}\sum_{n=0}^{\infty}\frac{1}{2n+1}^6 \times\left[2\delta-\frac{2+\exp[-(2n+1)^2\pi^2 D(\Delta-\delta)/a^2]-2\exp[-(2n+1)^2\pi^2 D\delta/a^2] -2\exp[-(2n+1)^2\pi^2 D\Delta/a^2]+\exp[-(2n+1)^2\pi^2 D(\Delta+\delta)/a^2]}{(2n+1)^2\pi^2 D/a^2}\right]\right)$
	C4:	$\exp\left(-8\pi^2 q^2\sum_{m=1}^{\infty}\frac{\left[2Da_m^2\delta-2+2e^{-Da_m^2\delta}+2e^{-Da_m^2\Delta}-e^{-Da_m^2(\Delta-\delta)}-e^{-Da_m^2(\Delta-\delta)}\right]}{\delta^2 D^2 a_m^6(R^2 a_m^2-1)}\right)$
	S4:	$\exp\left(\frac{2\gamma^2 G^2}{D}\sum_{m=1}^{\infty}\frac{a_m^{-4}}{a_m^2 R^2-2}\times\left[2\delta-\frac{2+e^{-a_m^2 D(\Delta-\delta)}-2e^{-a_m^2 D\delta}-2e^{-a_m^2 D\Delta}+e^{-a_m^2 D(\Delta+\delta)}}{a_m^2 D}\right]\right)$

2.3 Parametric Spatial Distributions

In Dmipy we can implement parametric spatial distributions on model parameters. **Gamma Distribution** The probability density of a Gamma distribution is given as

$$P_{\text{Gamma}}(x;\alpha,\beta)=\frac{\beta^\alpha x^{\alpha-1}e^{-x\beta}}{\Gamma(\alpha)} \tag{S16}$$

with $\Gamma(\alpha)$ a Gamma function.

3 GENERALIZED MODEL OPTIMIZATION

3.1 MC and MC-SM optimization

In terms of model fitting, the optimization of MC and MC-SM models are the same. The general shape of any of model composition of these types is given by

$$\mathbf{p}^*(\mathbf{x}) = \text{argmin}_{\mathbf{p}} \int \left[E(\mathbf{x}, \mathbf{A}) - \hat{E}^{\text{MC}}(\mathbf{A}, \mathbf{p}) \right]^2 d\mathbf{A}, \tag{S17}$$

$$\text{with } \hat{E}^{\text{MC}}(\mathbf{A}, \mathbf{p}) = \sum_i^N f_i C_i(\mathbf{A}, \mathbf{p}_i). \tag{S18}$$

So far, we have two implemented two optimizers that can optimize for these model types. First, we discuss the "brute2fine" optimizer that is a straight-forward implementation of brute force followed by local optimization. Second, we have the stochastic "mix" optimizer, based on the works by Farooq et al. (2016).

3.1.1 "brute2fine" optimization using Scipy's L-BFGS-B

As a standard optimizer Dmipy can use Scipy's open-source implementation of Low-memory Broyden–Fletcher–Goldfarb–Shanno with bounds (l-BFGS-b) (Byrd et al., 1995; Jones et al., 2001–). To summarize, the l-BFGS-b algorithm allows for the bounded minimization of arbitrary differentiable cost functions, as long as a starting point is provided. To impose a unity constraint on the volume fractions, we adopt the approach of Zhang et al. (2012), who nest the volume fractions of the multi-compartment model as

$$S(\tilde{g}, \mathbf{p}) = \tilde{f}_1 S_1(\tilde{g}, \mathbf{p}_1) + (1 - \tilde{f}_1)\tilde{f}_2 S_2(\tilde{g}, \mathbf{p}_2) + \ldots + (1 - \tilde{f}_1)\ldots(1 - f_{N-1}) S_n(\tilde{g}, \mathbf{p}_n) \tag{S19}$$

such that the nested volume volume fractions are all bounded by the closed unit interval $0 \leq \tilde{f}_i \leq 1$. Using this definition, we first use a straightforward brute-force algorithm to find an initial guess for the l-BFGS-b algorithm:

1. the signal is for *optimized* parameter combinations that are evenly sampled between the minimum and maximum bounds of each parameter, with the exception of orientation - in that case, we sample homogeneously on the sphere following the sampling approach by Caruyer et al. (2013).
2. the standard mean squared error is computed between the measured signal and all modeled signals, and the best fit is selected as the initial guess.

In step 1 we emphasize that the parameters are sampled evenly in the *optimized* parameter space, which can be different from the actual base-model parameter by introducing optimization parameters. Once the initial guess is given to the l-BFGS-b algorithm the Scipy-native implementation is used with all its standard optimization parameters. When the optimization finishes, the nested volume fractions are recomputed into standard ones, which due to the nesting now by definition add up to unity.

3.1.2 Microstructure Imaging in Crossings (MIX)

Recently, Farooq et al. (2016) proposed the microstructure in crossings (MIX) algorithm as a general framework for dMRI multi-compartment optimization. To explain the algorithm, let us separate all model parameters $\mathbf{p}$ into linear (volume fraction) parameters $\mathbf{f}$ and non-linear parameters $\mathbf{x}$, such that $\mathbf{p} = \{\mathbf{f}, \mathbf{x}\}$. The estimation of non-linear parameters $\mathbf{x}$ can be solved by stochastic search procedures, for which we use scipy's *differential evolution* implementation (Storn and Price, 1997). Once estimates of $\mathbf{x}$ are obtained, searching for $\mathbf{f}$ is a linear least-squares estimation problem. Summarizing the work of Farooq et al. (2016), the algorithm consists of 4 steps:

Step 1 - Variable Separation: We can then rewrite the problem in Eq (S17) as

$$\text{argmin}_{\mathbf{x},\mathbf{f}} ||E(\tilde{g}) - \phi(\tilde{g}, \mathbf{x})\mathbf{f}^T||_2^2 \tag{S20}$$

with $\phi(\tilde{g}, \mathbf{x}) = [C_1(\tilde{g}, \mathbf{x}_i), \ldots, C_{N_{models}}(\tilde{g}, \mathbf{x}_{N_{models}})] \in \mathbb{R}^{N_{DWI} \times N_{models}}$, with $\{\mathbf{x}_i\} \in \mathbf{x}$ being the non-linear parameters corresponding to each model i. It can be noted that

$$\mathbf{f} = \phi(\tilde{g}, \mathbf{x})^\dagger E(\tilde{g}) \tag{S21}$$

where $\phi^\dagger$ is the Moore-Penrose pseudo-inverse of ϕ. After the projection, the objective function takes the following form:

$$\text{argmin}_{\mathbf{x}} ||E(\tilde{g}) - \phi(\tilde{g}, \mathbf{x})\phi(\tilde{g}, \mathbf{x})^\dagger E(\tilde{g})||_2^2. \tag{S22}$$

Step 2 - Stochastic search for nonlinear parameters x: As we noted for l-BFGS-g, gradient-based methods require a good starting point to obtain an appropriate solution for the parameters. MIX, instead, uses stochastic optimization to overcome the need for an initial grid-search of the parameter space.

Step 3 - Constrained search for linear parameters f: Once an initial approximation of $\mathbf{x}^*$ is known, the third step is to obtain a constrained estimate of the linear parameters $\mathbf{f}$ such that they add up to one:

$$\text{argmin}_{\mathbf{f}} ||E(\tilde{g}) - \phi(\tilde{g}, \mathbf{x}^*)\mathbf{f}'||_2^2 \quad s.t. \sum_i \mathbf{f}_i = 1, \mathbf{f} \geq 0. \tag{S23}$$

We implement this step using scipy's Constrained Optimization BY Linear Approximation (COBYLA) algorithm.

Step 4 - Non-linear least squares estimation: The last step is to refine the solution by minimizing the standard objective function in Eq (S17) using the estimate of $\mathbf{x}^*$ and $\mathbf{f}^*$ as a starting point. For this, we again use the l-BFGS-b algorithm.

3.2 MC-SH Optimization

MC-SH modeling must be optimized differently than MC and MC-SM models, because only the spherical harmonics coefficients are estimated, with all other (non-linear) parameters fixed before any data fitting.

The general equation for MC-SH modeling and optimization is given by

$$\mathbf{c}^*(\mathbf{x}) = \text{argmin}_{\mathbf{c}} \int \left[E(\mathbf{x},\mathbf{A}_s) - \hat{E}^{\text{MC-SH}}(\mathbf{A},\mathbf{c}|\tilde{\mathbf{p}})\right]^2 d\mathbf{A} \quad \text{s.t.} \;\; \mathrm{F}_i(\mathbf{n};\mathbf{c}) \geq 0, \tag{S24}$$

$$\text{with } \hat{E}^{\text{MC-SH}}(\mathbf{A}_s,\mathbf{c}|\tilde{\mathbf{p}}) = \left[\sum_{i=1}^{N} \mathrm{F}_i(\mathbf{c}_i) \; *_{\mathbb{S}^2} \; C_i^K(\mathbf{A}_s|\tilde{\mathbf{p}}_i)\right](\mathbf{n}). \tag{S25}$$

In Dmipy, we implement two algorithms to can optimize MC-SH models. The first is the standard Constrained Spherical Deconvolution (CSD) algorithm by Tournier et al. (2007). The second is a generalized implementation of the multi-tissue CSD (MT-CSD) algorithm by Jeurissen et al. (2014).

3.2.1 CSD implementation by Tournier07

The classic Constrained Spherical Deconvolution (CSD) algorithm by Tournier et al. (2007) does not implement a hard constraint on a negative FOD, but instead penalizes negative values with a regularization matrix. Formally, their optimization is given by

$$\mathbf{c}^* = \text{argmin}_{\mathbf{c}} ||\mathbf{y} - \mathbf{M}^{\text{MS}}\mathbf{c}||^2 + \lambda|\mathbf{L}\mathbf{c}||^2 \tag{S26}$$

where $\mathbf{L}$ is a projection of $\mathbf{c}$ to the sphere like $\Phi\mathbf{c}$, but the matrix Φ is then adapted such that only lines are non-zero that project to a negative FOD value (or smaller than some threshold value). The minimum solution to this equation is simply

$$\mathbf{c}_i^* = (\mathbf{M}^T\mathbf{M} + \lambda\mathbf{L}^T\mathbf{L})\mathbf{M}^T\mathbf{y}, \tag{S27}$$

where at each iteration the matrix $\mathbf{L}$ is updated with the previous solution for $\mathbf{c}$, until the solution no longer moves. The initial condition for $\mathbf{c}_0$ is given by the non-constrained spherical deconvolution of order $l_{max} = 4$.

This implementation is not compatible for multiple compartments, but is entirely implemented using Numpy, meaning it's very fast. When only fitting a single compartments (or multiple compartments whose volume fractions are fixed), use this optimizer to solve MC-SH models.

3.2.2 MT-CSD implementation by Jeurissen14

The Multi-Tissue CSD implementation by Jeurissen et al. (2014) is exactly the equation in Eq. (S11). The problem can be directly given as input to the CVXPY optimization framework, which then returns our multi-compartment SH coeffients.

This optimizer can only be used when the model has at most 1 anisotropic convolution kernel. If multiple anisotropic kernels are given, the solution is very ill-posed as the two FOD distributions are too general.

4 ADDITIONAL MULTI-COMPARTMENT MODEL IMPLEMENTATIONS

Lastly, we have some extra MC-model implementations that were not demonstrated in the main paper, but we can easily constuct. The references for these models here are given in Table S3.

Table S3. Overview and composition of implemented MC-models and the number of lines that is needed to implement them from scratch using Dmipy, along with their primary reference. The MC-models above the midline are presented in the results section of this work, while the implementations of those below the midline are provided in the Supplementary Material.

Model Acronym	Model Composition	# of lines	Primary References
ActiveAx (Snippet 1)	G1+G2+S1+C4	13	(Alexander et al., 2010)
VERDICT (Snippet 2)	S4+G1+C1	8	(Panagiotaki et al., 2014)
SMT (Snippet 3)	G2	5	(Kaden et al., 2015)
SMT-NODDI (Snippet 4)	G1+SD3*(C1+G2)	21	(Cabeen et al., 2019)
CSD (Snippet 5)	TR2	4	(Tournier et al., 2007)

4.1 ActiveAx

Proposed by Alexander et al. (2010), the ActiveAx model models the generalized white matter composition as a combination of a parallel bundle consisting of a Cylinder and Zeppelin with tortuosity constraint, a non-diffusing Dot compartment and a Ball for CSF contributions. Formally, the model is given by

$$E_{\text{ActiveAx}} = \underbrace{f_{\text{CSF}}\overbrace{G1(\cdot|\lambda_{\text{iso}})}^{\text{Ball}}}_{\text{CSF}} + \underbrace{f_{\text{trap}}\times\overbrace{S1}^{\text{Dot}}}_{\text{Trapped Water}} + \underbrace{f_h\overbrace{G2(\boldsymbol{\mu}|\lambda_{\|},\lambda_{\perp}^{\text{tort}})}^{\text{Zeppelin}}}_{\text{Hindered Extra-Axonal}} + \underbrace{f_r\overbrace{C4(\boldsymbol{\mu}\langle D\rangle|\lambda_{\|},\lambda_{\perp})}^{\text{Cylinder}}}_{\text{Intra-Axonal}}, \tag{S28}$$

where on practice only the axon diameter $\langle D\rangle$, bundle orientation $\boldsymbol{\mu}$, and the volume fractions are estimated. Dmipy's implementation of ActiveAx is given in Snippet 1.

```
from dmipy.core import modeling_framework
from dmipy.distributions import distribute_models
from dmipy.signal_models import gaussian_models, cylinder_models, sphere_models
ball = gaussian_models.G1Ball()
cylinder = cylinder_models.C4CylinderGaussianPhaseApproximation()
dot = sphere_models.S1Dot()
zeppelin = gaussian_models.G2Zeppelin()
bundle = distribute_models.BundleModel([cylinder,zeppelin])
bundle.set_equal_parameter('C4CylinderGaussianPhaseApproximation_1_lambda_par', 'G2Zeppelin_1_lambda_par')
bundle.set_tortuous_parameter(
    'G2Zeppelin_1_lambda_perp', 'C4CylinderGaussianPhaseApproximation_1_lambda_par', 'partial_volume_0')
bundle.set_fixed_parameter('C4CylinderGaussianPhaseApproximation_1_lambda_par', 1.7e-9)
activax = modeling_framework.MultiCompartmentModel([ball, dot, bundle])
activax.set_fixed_parameter('G1Ball_1_lambda_iso', 3e-9)
```

Snippet 1: Dmipy Implementation of ActiveAx model in 13 lines.

4.2 VERDICT

Panagiotaki et al. (2014) proposed a multi-compartment model called VERDICT to characterize the composition of tumorous tissues. VERDICT models the diffusion in tumor cells, the extra-cellular space and surrounding bloodvessels as a restricted Sphere, an isotropic Gaussian Ball and a Stick compartment, respectively. VERDICT's design is as follows:

$$E_{\text{VERDICT}} = \underbrace{f_{\text{Tumor}}\overbrace{S4(D|\lambda_{\text{intra}})}^{\text{Sphere}}}_{\text{Tumor Cells}} + \underbrace{f_{\text{extra}}\overbrace{G1(\cdot|\lambda_{iso})}^{\text{Ball}}}_{\text{Hindered Extra-Cellular}} + \underbrace{f_{blood}\overbrace{C1(\lambda_{||},\boldsymbol{\mu})}^{\text{Stick}}}_{\text{Vascular}} \tag{S29}$$

where D is the sphere's diameter. VERDICT uses the Gaussian Phase approximation to model the sphere (Balinov et al., 1993), which accounts to changes in gradient pulse duration δ and separation Δ. Furthermore, some particular parameter constraints are imposed:

- The intra-cellular (intra-spherical) diffusivity is fixed to 0.9×10^{-9} m^2/s,
- The extra-cellular Gaussian diffusivity is also fixed to 0.9×10^{-9} m^2/s,
- The optimization range for $\lambda_{||}$ of the Vascular Stick is set between (3.05-10)$\times10^{-9}$ m^2/s.

We provide the Dmipy implementation of the VERDICT model in Snippet 2.

```
from dmipy.signal_models import sphere_models, cylinder_models, gaussian_models
from dmipy.core.modeling_framework import MultiCompartmentModel
sphere = sphere_models.S4SphereGaussianPhaseApproximation(diffusion_constant=0.9e-9)
ball = gaussian_models.G1Ball()
stick = cylinder_models.C1Stick()
verdict_mod = MultiCompartmentModel(models=[sphere, ball, stick])
verdict_mod.set_fixed_parameter('G1Ball_1_lambda_iso', 0.9e-9)
verdict_mod.set_parameter_optimization_bounds('C1Stick_1_lambda_par', [3.05e-9, 10e-9])
```

Snippet 2: VERDICT model in 8 lines of code.

4.3 SMT

The first spherical mean technique (SMT) model was proposed by Kaden et al. (2015). It is a simple MC-model with just a single Zeppelin, fitted in an MC-SM model. In this sense, it only first two parameters, $\lambda_{||}$ and $\lambda_{\perp}$, with $\lambda_{\perp} \leq \lambda_{||}$. The code to implement the SMT model is given in Snippet 3.

4.4 SMT-NODDI

In recent works, Cabeen et al. (2019) proposed to speed up the NODDI estimation time by splitting the optimization in two steps. In the first step, the NODDI model is fitted in the *spherical mean* sense, meaning only the volume fractions are estimated, and the orientation and dispersion influence should be obviated in this setting. In the second step, the volume fractions are fixed, and only the orientation and dispersion parameters are estimated.

```python
from dmipy.signal_models import gaussian_models
from dmipy.core.modeling_framework import MultiCompartmentSphericalMeanModel
zeppelin = gaussian_models.G2Zeppelin()
smt_mod = MultiCompartmentSphericalMeanModel(models=[zeppelin])
smt_mod.set_fractional_parameter('G2Zeppelin_1_lambda_perp', 'G2Zeppelin_1_lambda_par')
```

Snippet 3: SMT model in 5 lines of code.

It should be noted that the SMT-NODDI model is not equivalent to the standard NODDI model. The volume fractions will have different values, especially in crossing areas where the standard NODDI model would suffer due to fitting multiple bundles with a single bundle, but the SMT-NODDI model would be capable of handeling this in at least the first step. The implementation of the NODDI-SMT model is given in Snippet 4.

```python
from dmipy.signal_models import cylinder_models, gaussian_models
from dmipy.distributions.distribute_models import BundleModel, SD1WatsonDistributed
from dmipy.core import modeling_framework
# setup base CompartmentModels
stick = cylinder_models.C1Stick()
zeppelin = gaussian_models.G2Zeppelin()
ball = gaussian_models.G1Ball()
# setup distributed models and apply same parameter links
bundle = BundleModel([stick, zeppelin])
watson_bundle = SD1WatsonDistributed([stick, zeppelin])
for dist_mod in [bundle, watson_bundle]:
    dist_mod.set_tortuous_parameter('G2Zeppelin_1_lambda_perp', 'C1Stick_1_lambda_par','partial_volume_0')
    dist_mod.set_equal_parameter('G2Zeppelin_1_lambda_par', 'C1Stick_1_lambda_par')
    dist_mod.set_fixed_parameter('G2Zeppelin_1_lambda_par', 1.7e-9)
# setup mc-models and apply same fixed isotropic diffusivity
smt_noddi_mod = modeling_framework.MultiCompartmentSphericalMeanModel(models=[bundle, ball])
noddi_mod = modeling_framework.MultiCompartmentModel(models=[watson_bundle, ball])
for mc_mod in [smt_noddi_mod, noddi_mod]:
    mc_mod.set_fixed_parameter('G1Ball_1_lambda_iso', 3e-9)
# Step 1: fit SMT-NODDI model
smt_noddi_fit = smt_noddi_mod.fit(scheme, data)
# Step 2: fix volume fractions of SMT-NODDI in standard NODDI, and fit remaining NODDI parameters
noddi_mod.set_fixed_parameter('SD1WatsonDistributed_1_partial_volume_0',
                              smt_noddi_fit.fitted_parameters['BundleModel_1_partial_volume_0'])
noddi_mod.set_fixed_parameter('partial_volume_0',
                              smt_noddi_fit.fitted_parameters['partial_volume_0'])
noddi_mod.set_fixed_parameter('partial_volume_1',
                              smt_noddi_fit.fitted_parameters['partial_volume_1'])
noddi_fit_2step = noddi_mod.fit(scheme, data)
```

Snippet 4: 2-step SMT-NODDI implementation in 21 lines (excluding comments).

4.5 CSD

The original CSD model only contains a single anisotropic tissue response model that is directly estimated from the data. The original work obtained the response model by means of an FA mask on the data (Tournier et al., 2007). In this example, we obtain all three tissue responses (WM, GM, CSF) using the heuristic algorithm by Dhollander et al. (2016), and create the basic CSD model by just taking the WM response in Snippet 5.

```
from dmipy.core.modeling_framework import MultiCompartmentSphericalHarmonicsModel
from dmipy.tissue_response.three_tissue_response import three_tissue_response_dhollander16
wm, gm, csf, selection_map = three_tissue_response_dhollander16(
    scheme_hcp, data_hcp, wm_algorithm='tournier13',
    wm_N_candidate_voxels=150, gm_perc=0.2, csf_perc=0.4)
mt_csd_mod = MultiCompartmentSphericalHarmonicsModel(models=[wm])
```

Snippet 5: CSD model in 4 lines of code.

REFERENCES

Alexander, D. C., Hubbard, P. L., Hall, M. G., Moore, E. A., Ptito, M., Parker, G. J., et al. (2010). Orientationally invariant indices of axon diameter and density from diffusion mri. *Neuroimage* 52, 1374–1389

Assaf, Y., Freidlin, R. Z., Rohde, G. K., and Basser, P. J. (2004). New modeling and experimental framework to characterize hindered and restricted water diffusion in brain white matter. *Magnetic Resonance in Medicine* 52, 965–978

Balinov, B., Jonsson, B., Linse, P., and Soderman, O. (1993). The nmr self-diffusion method applied to restricted diffusion. simulation of echo attenuation from molecules in spheres and between planes. *Journal of Magnetic Resonance, Series A* 104, 17–25

Bingham, C. (1974). An antipodally symmetric distribution on the sphere. *The Annals of Statistics* , 1201–1225

Byrd, R. H., Lu, P., Nocedal, J., and Zhu, C. (1995). A limited memory algorithm for bound constrained optimization. *SIAM Journal on Scientific Computing* 16, 1190–1208

Cabeen, R., Sepehrband, F., and Toga, A. (2019). Rapid and accurate noddi parameter estimation with the spherical mean technique. In *ISMRM 2019*

Callaghan, P. T. (1995). Pulsed-gradient spin-echo nmr for planar, cylindrical, and spherical pores under conditions of wall relaxation. *Journal of magnetic resonance, Series A* 113, 53–59

Caruyer, E., Lenglet, C., Sapiro, G., and Deriche, R. (2013). Design of multishell sampling schemes with uniform coverage in diffusion mri. *Magnetic Resonance in Medicine* 69, 1534–1540

Descoteaux, M., Angelino, E., Fitzgibbons, S., and Deriche, R. (2007). Regularized, fast, and robust analytical q-ball imaging. *Magnetic Resonance in Medicine* 58, 497–510

Dhollander, T., Raffelt, D., and Connelly, A. (2016). Unsupervised 3-tissue response function estimation from single-shell or multi-shell diffusion mr data without a co-registered t1 image. In *ISMRM Workshop on Breaking the Barriers of Diffusion MRI*. vol. 5

Farooq, H., Xu, J., Nam, J. W., Keefe, D. F., Yacoub, E., Georgiou, T., et al. (2016). Microstructure imaging of crossing (mix) white matter fibers from diffusion mri. *Scientific reports* 6, 38927

Jeurissen, B., Tournier, J.-D., Dhollander, T., Connelly, A., and Sijbers, J. (2014). Multi-tissue constrained spherical deconvolution for improved analysis of multi-shell diffusion mri data. *NeuroImage* 103, 411–426

[Dataset] Jones, E., Oliphant, T., Peterson, P., et al. (2001–). SciPy: Open source scientific tools for Python. [Online; accessed ¡today¿]

Kaden, E., Knösche, T. R., and Anwander, A. (2007). Parametric spherical deconvolution: inferring anatomical connectivity using diffusion mr imaging. *NeuroImage* 37, 474–488

Kaden, E., Kruggel, F., and Alexander, D. C. (2015). Quantitative mapping of the per-axon diffusion coefficients in brain white matter. *Magnetic resonance in medicine*

Murday, J. and Cotts, R. M. (1968). Self-diffusion coefficient of liquid lithium. *The Journal of Chemical Physics* 48, 4938–4945

Panagiotaki, E., Walker-Samuel, S., Siow, B., Johnson, S. P., Rajkumar, V., Pedley, R. B., et al. (2014). Noninvasive quantification of solid tumor microstructure using verdict mri. *Cancer research*

Söderman, O. and Jönsson, B. (1995). Restricted diffusion in cylindrical geometry. *Journal of Magnetic Resonance, Series A* 117, 94–97

Storn, R. and Price, K. (1997). Differential evolution–a simple and efficient heuristic for global optimization over continuous spaces. *Journal of global optimization* 11, 341–359

Tournier, J.-D., Calamante, F., and Connelly, A. (2007). Robust determination of the fibre orientation distribution in diffusion mri: non-negativity constrained super-resolved spherical deconvolution. *NeuroImage* 35, 1459–1472

Vangelderen, P., DesPres, D., Vanzijl, P., and Moonen, C. (1994). Evaluation of restricted diffusion in cylinders. phosphocreatine in rabbit leg muscle. *Journal of Magnetic Resonance, Series B* 103, 255–260

Zhang, H., Schneider, T., Wheeler-Kingshott, C. A., and Alexander, D. C. (2012). Noddi: practical in vivo neurite orientation dispersion and density imaging of the human brain. *Neuroimage* 61, 1000–1016
